# ERα-LBD, an isoform of estrogen receptor alpha, promotes breast cancer proliferation and endocrine resistance

**DOI:** 10.1038/s41523-022-00470-6

**Published:** 2022-08-23

**Authors:** Antonio Strillacci, Pasquale Sansone, Vinagolu K. Rajasekhar, Mesruh Turkekul, Vitaly Boyko, Fanli Meng, Brian Houck-Loomis, David Brown, Michael F. Berger, Ronald C. Hendrickson, Qing Chang, Elisa de Stanchina, Fresia Pareja, Jorge S. Reis-Filho, Ramya Segu Rajappachetty, Isabella Del Priore, Bo Liu, Yanyan Cai, Alex Penson, Chiara Mastroleo, Marjan Berishaj, Francesca Borsetti, Enzo Spisni, David Lyden, Sarat Chandarlapaty, Jacqueline Bromberg

**Affiliations:** 1 Department of Medicine, Memorial Sloan Kettering Cancer Center, New York, NY USA; 2 Human Oncology and Pathogenesis Program, Memorial Sloan Kettering Cancer Center, New York, NY USA; 3 Department of Biological, Geological and Environmental Sciences, University of Bologna, Bologna, Italy; 4 Children’s Cancer and Blood Foundation Laboratories, Weill Cornell Medicine, New York, NY USA; 5 Department of Surgery, Memorial Sloan Kettering Cancer Center, New York, NY USA; 6 Molecular Cytology Core Facility, Memorial Sloan Kettering Cancer Center, New York, NY USA; 7 Center for Molecular Oncology, Memorial Sloan Kettering Cancer Center, New York, NY USA; 8 Microchemistry and Proteomics, Memorial Sloan Kettering Cancer Center, New York, NY USA; 9 Antitumor Assessment Core Facility, Memorial Sloan Kettering Cancer Center, New York, NY USA; 10 Department of Pathology, Memorial Sloan Kettering Cancer Center, New York, NY USA; 11 Cancer Biology and Genetics Program, Memorial Sloan Kettering Cancer Center, New York, NY USA; 12 Department of Pharmacy and Biotechnology, University of Bologna, Bologna, Italy; 13 Department of Medicine, Weill Cornell Medicine, New York, NY USA

**Keywords:** Breast cancer, Cancer stem cells, Transcriptomics

## Abstract

Estrogen receptor alpha (ERα) drives mammary gland development and breast cancer (BC) growth through an evolutionarily conserved linkage of DNA binding and hormone activation functions. Therapeutic targeting of the hormone binding pocket is a widely utilized and successful strategy for breast cancer prevention and treatment. However, resistance to this endocrine therapy is frequently encountered and may occur through bypass or reactivation of ER-regulated transcriptional programs. We now identify the induction of an ERα isoform, ERα-LBD, that is encoded by an alternative *ESR1* transcript and lacks the activation function and DNA binding domains. Despite lacking the transcriptional activity, ERα-LBD is found to promote breast cancer growth and resistance to the ERα antagonist fulvestrant. ERα-LBD is predominantly localized to the cytoplasm and mitochondria of BC cells and leads to enhanced glycolysis, respiration and stem-like features. Intriguingly, ERα-LBD expression and function does not appear to be restricted to cancers that express full length ERα but also promotes growth of triple-negative breast cancers and ERα-LBD transcript (ESR1-LBD) is also present in BC samples from both ERα(+) and ERα(−) human tumors. These findings point to ERα-LBD as a potential mediator of breast cancer progression and therapy resistance.

## Introduction

The nuclear receptor estrogen receptor alpha (ERα) is the major therapeutic target for the ~75% of breast cancers (BC) where its expression is detected^1^. ERα is a multifunctional protein that contributes to cellular processes via transcriptional regulation, participation in signaling complexes^2,3^ and regulation of mitochondrial function^4,5^. Its function in primary BC growth depends mainly on its activation of transcription in response to hormone binding-mediated conformational changes in the receptor. Thus, therapeutic targeting of ERα has been directed at inhibiting this hormone-receptor interaction. The major forms of therapy include suppressors of estrogen biosynthesis (e.g. aromatase inhibitors, GNRH antagonists) and direct ERα antagonists (e.g. tamoxifen, fulvestrant)^6–8^. Cancers develop resistance to these forms of therapy over time, often through alterations in the *ESR1* gene including activating point mutations in the ligand binding domain and gene fusions^9–12^ that restore ERα transcriptional activity. In addition, cancers can bypass ERα signaling and activate oncogenic functions through genetic alterations that involve other growth factor signaling pathways, modification of cell cycle regulators and increases in stem cell activity^13–17^. In addition, epigenetic mechanisms of resistance may also be contributory and whether these can affect ERα or these other growth pathways is a key question, particularly given that up to half of all resistant tumors harbor no mutation known to cause resistance^13,18^.

About 15% of BC cases are triple-negative breast cancers (TNBCs), lacking the expression of ERα, PR and HER2-amplification and are thus not responsive to targeted therapies against ERα or HER2. These cancers are associated with a poorer prognosis due to a higher rate of metastatic progression and resistance to treament^19,20^. Notably, TNBC may be responsive to estrogen stimulation via ERα-independent pathways, promoting tumor formation and progression via different molecular mechanisms^21^.

In this work, we investigated ERα protein and transcripts in *ESR1* wild-type breast cancers that were resistant to fulvestrant and in TNBC. We identified an isoform that maintains the ligand binding domain but lacks the DNA binding domain, and promotes breast cancer growth and endocrine resistance through non-canonical functions outside the nucleus. These data reinforce the importance of the broader biological functions of ER protein family outside of its transcriptional activation role.

## Results

### Fulvestrant resistant cells express an estrogen receptor α isoform

**Fig. 1:**
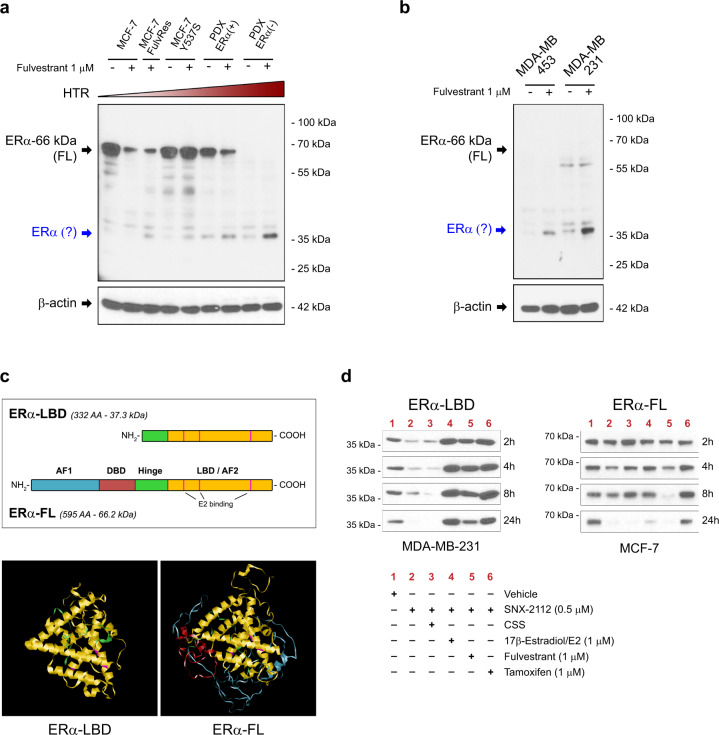
Fulvestrant resistant BC cell lines express ERα-LBD, a novel estrogen receptor α isoform. **a**, **b** Western blot analysis of estrogen receptor alpha (ERα) protein expression in breast cancer (BC) cell lines. Cells were cultured for 24 h in the presence of vehicle (−) or fulvestrant 1 µM (+) before lysis. Vehicle = DMSO. ERα protein expression was normalized against β-actin. Increasing levels of hormonal therapy resistance (HTR) are indicated. A potential smaller ERα isoform is indicated in blue. **c** Comparison between ERα-LBD and ERα-FL (full-length): domains (AF-1, transcription Activation Function-1; DBD, DNA Binding Domain; Hinge; LBD, Ligand Binding Domain; AF-2, transcription Activation Function-2), amino acid (AA) number, molecular weight, 3-D structure prediction (Phyre2). **d** ERα protein stability assay. ERα-LBD and ERα-FL protein levels were analyzed by western blot in MDA-MB-231 and MCF-7 cells, respectively. Protein samples were collected at different time points (2-24 h), after treatment. Physiological levels of E2 (pM range) were present in all samples, except samples in lane 3 and 4. Vehicle DMSO, CSS charcoal-stripped serum. Source data are provided as a Source Data file.

In order to investigate novel mechanisms of hormonal therapy resistance (HTR) in breast cancer, we examined the expression of ERα protein in different BC cell lines, either in the absence or presence of the selective estrogen receptor degrader (SERD) fulvestrant (ICI 182,780) which reduces ERα full-length (ERα-FL, 66 kDa) protein levels as a consequence of reduced stability and dimerization^22,23^. We postulated that changes in fulvestrant-mediated suppression of ERα-FL levels may promote drug resistance and investigated this response in several models selected for fulvestrant resistance (MCF-7 FulvRes, MCF-7 Y537S, two breast cancer PDX models, and T-47D FulvRes)^17,24^, compared to fulvestrant-sensitive cells (Fig. 1a and Supplementary Fig. 1a, b). After 24 h exposure to 1 µM fulvestrant, MCF-7 cells, and MCF-7 FulvRes showed ~80% and ~60% loss of ERα-FL expression, respectively; T-47D and T-47D FulvRes showed a ~60% and ~30% reduction respectively; MCF-7 Y537S and PDX-ERα(+) showed no decrease or slight decrease (~30%), respectively. PDX-ERα(−) showed no expression at all. Notably, we observed a faster-migrating band (~37 kDa) in all four resistant models that appeared to increase in response to fulvestrant. As this protein was detected in both ERα positive and negative models, we analyzed by immunoblotting two triple-negative breast cancer (TNBC) cell lines, MDA-MB-453 and MDA-MB-231, also lacking ERα-FL expression and therefore resistant to fulvestrant treatment (Supplementary Fig. 1a). Importantly, both TNBC cell lines displayed this lower molecular weight protein whose expression increased after fulvestrant treatment (Fig. 1b). The specificity of the ERα-related ~37 kDa band was confirmed by using two different antibodies both raised against the C-terminus of ERα protein (Fig. 1a, b and Supplementary Fig. 1c). To establish the identity of the protein, we performed immunoprecipitation and mass spectrometry experiments from MCF-7 FulvRes and MDA-MB-231 cells and determined that the ERα-related protein is a truncated isoform of ERα-FL, lacking the N-terminal domains AF1 (transcription Activation Function-1), DBD (DNA Binding Domain) and a portion of the hinge domain, and it is composed principally by the C-terminal domains LBD (Ligand Binding Domain) and AF2 (Fig. 1c and Supplementary Fig. 1d). Based on our observation, we named this ERα protein isoform ‘ERα-LBD’. ERα-LBD has 332 amino acids, a MW of 37.3 kDa, and a predicted 3-D structure that is similar to the predicted ERα-FL (Fig. 1c and Supplementary Fig. 1e). As expected, we failed to detect a 37.3 kDa protein using an ERα antibody raised against the N-terminus (Supplementary Fig. 1f).

Across all the models we investigated, we found that fulvestrant increased expression of ERα-LBD and in some cases was necessary for detection. These data suggested that fulvestrant was serving to stabilize ERα-LBD akin to the way ligands protect full-length ER against proteolytic degradation when unchaperoned by HSP90. To further address this possibility, we analyzed ERα-LBD and ERα-FL expression upon HSP90 inhibition using an ATP inhibitor of HSP90 function (SNX-2112)^25,26^. Following SNX-2112 treatment, both ERα-FL and ERα-LBD underwent degradation, either in the presence (lane 2, media supplemented with regular 10% FBS) or absence (lane 3, media supplemented with 10% charcoal-stripped FBS) of physiological E2 levels (pM range). Although, ERα-LDB degradation was more rapid, especially in the absence of E2 (lane 3, CSS). Conversely, high levels of E2, fulvestrant or tamoxifen treatment (1 µM) led to increased stability of ERα-LBD. (Fig. 1d and Supplementary Fig. 1g, h).

Taken together, our data demonstrate that ERα(+) and (−) BC models characterized by HTR can produce a truncated ERα isoform whose expression can be induced by ERα ligand binding pocket compounds, including fulvestrant.

### ERα-LBD is encoded by an ESR1 transcript variant identified in both cell lines and primary breast cancers

**Fig. 2:**
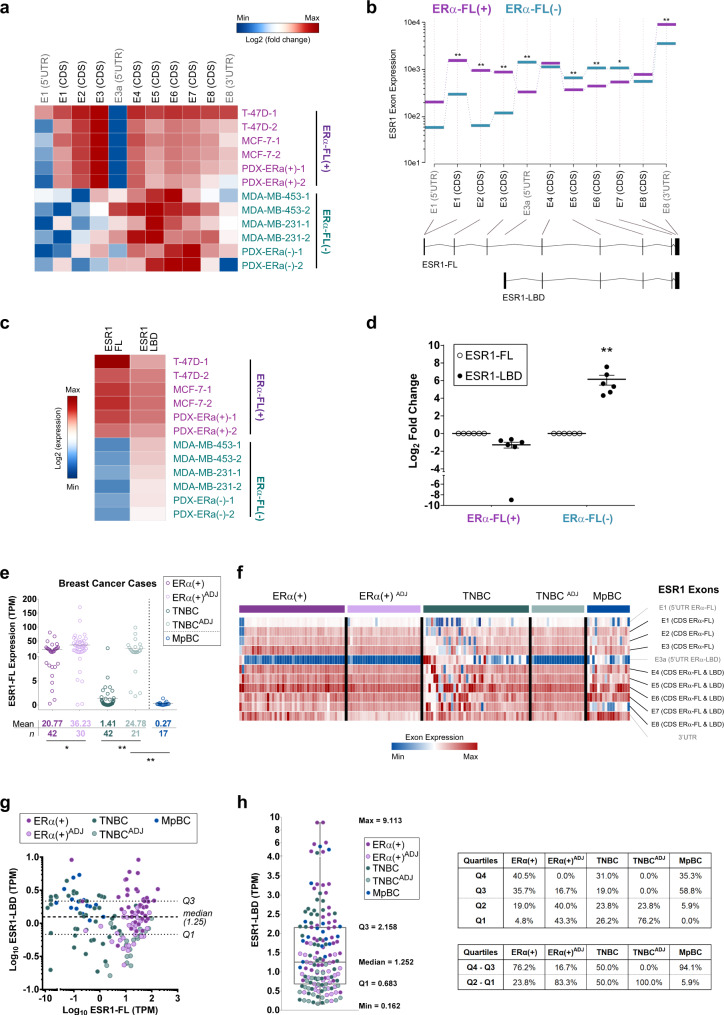
ERα-LBD is encoded by an ESR1 transcript variant. **a** Heat map showing ESR1 exon expression in BC cell lines, calculated by using DEXSeq-count tool (log2, mean-centered). Exons encoding ERα-FL protein are indicated in black (CDS). BC cell lines were grouped as ERα-FL(+), including T-47D, MCF-7, PDX-ERα(+) (in purple), and ERα-FL(−), including MDA-MB-453, MDA-MB-231, PDX-ERα(−) (in teal). **b** Differential ESR1 exon expression between ERα-FL(+) and ERα-FL(−) groups was calculated using DEXSeq and plotted using plotDEXSeq tools. Exons associated with two different ESR1 transcript variants were taken under consideration for the analysis: ESR1-FL (main variant encoding ERα-FL) and ESR1-LBD (predicted, encoding ERα-LBD). Expression values for ERα-FL(+) and ERα-FL(−) groups are plotted in purple and in teal, respectively. BH adjusted *P*-values (FDR test): *P < 0.05, **P < 0.01 (*n* = 6/group). **c** Heat map showing ESR1 transcript (ESR1-FL and ESR1-LBD) expression among BC cell lines, grouped as ERα-FL(+) (purple) and ERα-FL(−) (teal). Transcript expression was calculated by using Kallisto tool and quantified as counts/transcript length. **d** ESR1-LBD transcript expression plotted as log2 fold change, relative to ESR1-FL. ESR1-FL, empty circles; ESR1-LBD, solid circles. Mean ± s.e.m. is shown (*n* = 6); **P < 0.01, ratio paired *t* test, two-sided. **e** ESR1-FL transcript expression was calculated in BC samples by using Kallisto and plotted as Transcripts Per Kilobase Million (TPM). ERα(+) BC primary tumors (*n* = 42); uninvolved breast tissue adjacent to ERα(+) primary tumors (*n* = 30); TNBC primary tumors (*n* = 42); uninvolved breast tissue adjacent to TNBC primary tumors (*n* = 21); metaplastic BC primary tumors (MpBC, *n* = 17). **f** Heat map showing ESR1 exon expression in BC patient groups. Exon expression was calculated by using DEXSeq-count tool [# of read counts/exon length] and scaled by column. **g** Dispersion plot for ESR1 transcript variants expression (ESR1-FL vs. ESR1-LBD), calculated as TPM. Dashed lines corresponding to ESR1-LBD expression median and Q1**-**Q3 quartiles are shown. Color code as in **e**. **h** Box and whiskers plot representing the distribution of ESR1-LBD expression values (TPM) from BC samples. Median, Q1/Q3 and Min/Max values are shown. Color code as in **e**. Percentage of samples from each group is also shown, distributed into quartiles. Source data are provided as Supplementary Data 2 file.

To understand the basis of ERα-LBD formation we carried out RNA capture-sequencing and PCR assays to analyze the usage of ESR1 exons and transcripts in different models, including both ERα-FL positive and negative cells. High-depth capture-sequencing data showed that BC ERα-FL(−) cells expressing exclusively ERα-LBD, such as MDA-MB-453/-231 and PDX-ERα, have higher abundance of ESR1 read counts in the C-terminus (CDS exons #4, 5, 6, 7 and 8), compared to the N-terminus (CDS exons #1–3). In contrast, ESR1 exon usage profile was significantly different in ERα-FL(+) cells, such as T-47D, MCF-7, and PDX-ERα(+), where the expression of exons #1–3 was higher than exons #4–8 (Fig. 2a and Supplementary Data 1). To identify an ESR1 transcript variant for ERα-LBD, we searched the FANTOM CAT (CAGE Associated Transcriptome) database, which displayed an additional ESR1 exon between exons #3 and #4, with a transcription start site (TSS) at the 5′ end (Supplementary Fig. 2a)^27,28^. We annotated this region as 5′UTR of a putative ESR1-LBD transcript variant (exon E3a). In this region, a higher number of read counts was found in ERα(−) cells, compared to ERα(+) (Fig. 2a). Differential exon usage was determined for ESR1-FL (encoding ERα-FL) and a predicted ESR1-LBD (encoding ERα-LBD) transcript by comparing ERα-FL(+) and ERα(−) groups, revealing a significantly lower usage of exons E1, E2, E3 and E8-3UTR, and higher usage of exons E3a, E5, E6, and E7 for the ERα(−) group (Fig. 2b).

Exon data were supported by the analysis of ESR1 transcripts expression, demonstrating higher levels of ESR1-LBD transcript in ERα-FL(−) cells, compared to ESR1-FL (Fig. 2c, d and Supplementary Data 1). Also, qPCR (exon ‘walking’) and RT-PCR assays confirmed the RNA-seq results. ERα-FL(+) and ERα-FL(−) cells express an ESR1 mRNA ranging from exon 1 to 8 and from exon 4 to exon 8, respectively (Supplementary Fig. 2b, c). These results are in accordance with the western blot data in Fig. 1b, showing that ERα-FL(−) cells express a truncated ERα protein (LBD) and not full-length ERα. As definitive proof, the stable cloning of the putative ERα-LBD CDS into BC cells (exon 4 to exon 8), led to ERα-LBD overexpression (oe) whereas targeted genomic deletions within exon 4 (by CRISPR technology) induced ERα-LBD knockdown (kd) (Supplementary Fig. 2d). Alignment studies performed on public databases (UCSC Genome Browser) showed that the genomic region surrounding exon E3a is characterized by high-density transcription binding sites and candidate cis-regulatory elements (Supplementary Fig. 2e). Thus, we collected preliminary data based on luciferase-reporter assays to support the hypothesis of a putative alternative ESR1 promoter within exon E3a genomic region. Our data demonstrated the presence of transcriptional activity within the ESR1-LBD 5′UTR region (E3a) in both ERα(+) and ERα(−) cell lines (Supplementary Fig. 2f).

RNA sequencing and qPCR data showed reduced expression of the ESR1 3′UTR sequence in ERα-FL(−) cells as compared to ERα-FL(+) ones (Fig. 2a, b and Supplementary Fig. 2b). It has been observed that shortening of 3′UTRs by alternative cleavage and polyadenylation is associated with increased RNA stability, and that this phenomenon can promote oncogene mediated transformation by enhanced protein production^29^. Thus, we hypothesized that the partial/total loss of the 3′UTR sequence in ERα-FL(−) cells may enhance ESR1 mRNA stability. To test this, we treated BC cells with actinomycin-D (inhibitor of RNA synthesis) and indeed the LBD portion of ESR1 transcript was significantly more stable than the FL-specific in MCF-7 FulvRes and TNBC cells (Supplementary Fig. 2g).

We note that ERα-LBD is not the only ERα transcript variant that has been observed in breast cancer. An ERα-36 variant, encoded by ESR1 exons #2–6 and an additional exon downstream of the *ESR1* gene, encoding a protein expressing the DNA-binding domain, a truncated LBD and a partial dimerization domain has been described in a variety of cancer models including tamoxifen-resistant breast cancers^30,31^. Importantly, ERα-LBD is distinct from ERα-36 as specific primers and an antibody for the ERα-36 isoform failed to detect ERα-LBD (Supplementary Fig. 2h, i).

In addition, we examined ESR1-FL /-LBD expression by analyzing RNA-seq samples from human BC specimens. ESR1-FL overall expression was assessed on the following collection of samples: ERα(+) BC primary tumors (*n* = 42); uninvolved breast tissue adjacent to ERα(+) primary tumors (*n* = 30); TNBC primary tumors (*n* = 42); uninvolved breast tissue adjacent to TNBC primary tumors (*n* = 21); metaplastic BC primary tumors (MpBC, *n* = 17)^32,33^. As expected, ESR1-FL transcript was found to be markedly reduced in TNBC and MpBC samples (Fig. 2e). Notably, ERα expression is known to be present in normal breast epithelium^34^. For each cancer sample, ESR1 exon expression was also calculated and, similarly to what we observed with BC cell lines (Fig. 2a), TNBC and MpBC samples showed higher amounts of 5′UTR exon 3a and CDS exons #4–8, compared to #1–3 (Fig. 2f). We then examined the levels of the ESR1-LBD transcript in BC groups, relative to ESR1-FL (Fig. 2g). 76% of ERα(+), 50% of TNBC, and 81% of MpBC were characterized by high ESR1-LBD levels (Q3 + Q4 quartiles, above the median calculated on all samples). Interestingly, only 17% of ERα(+)^ADJ^ and none of TNBC^ADJ^ samples were found to have high ESR1-LBD expression (Fig. 2h). All expression data are summarized in Supplementary Data 2.

### ERα-LBD localizes to the mitochondria and cytoplasm of BC cells

**Fig. 3:**
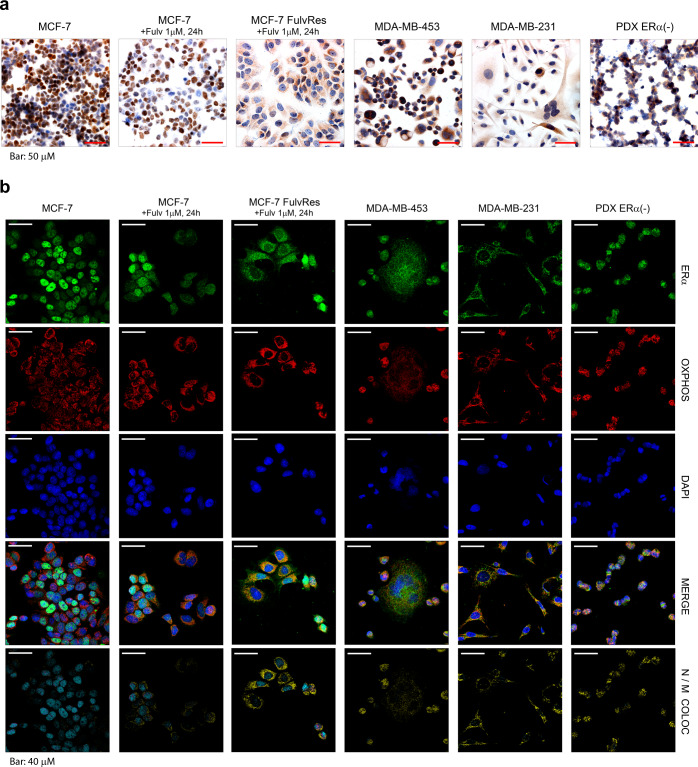
ERα-LBD localizes in the cytoplasm and mitochondria of breast cancer cells. **a** Representative images of ERα staining by immunohistochemistry (IHC) in six different BC cell lines. All histological sections were counterstained with hematoxylin. Scale bar representing 50 μm is shown. Magnification: ×40. **b** Representative confocal images of BC cells stained for ERα (green), mitochondria/OXPHOS (red) and nuclei/DAPI (blue). For each cell line, merged images and colocalization images are also shown. Color code for colocalization: cyan (ERα-Nuclei, N); yellow (ERα-Mitochondria, M). A scale bar for each image representing 40 μm is shown. Magnification: ×63.

ERα-LBD lacks the N-terminal domain of the full-length receptor and we thus hypothesized it may have a distinctive cellular location. Using ERα immunohistochemistry (IHC), full-length expressing MCF-7 cells show robust nuclear staining that is markedly reduced 24 h after fulvestrant treatment. By contrast, ERα-LBD expressing models MCF-7 FulvRes, MDA-MB-453/-231, and PDX-ERα(−) display punctate cytoplasmic foci (Fig. 3a). Based on this particular pattern, we hypothesized that ERα-LBD might be localized to the mitochondria and performed confocal analyses examining cells for ERα protein (green), OXPHOS (mitochondrial marker, red) and DAPI (nuclear marker, blue) (Fig. 3b). We examined images from both separate and merged channels, and by 3-D image processing to evaluate the co-localization volume between ERα, nuclei or mitochondria. The overall IF staining confirmed data from the IHC: MCF-7 cells have a strong ERα nuclear localization which is decreased after fulvestrant treatment; MCF-7 FulvRes chronically treated with fulvestrant have both nuclear and mitochondrial ERα staining. In ERα full-length negative cells, ERα-LBD was largely absent from the nucleus and found in the cytoplasm with a substantial fraction co-localized with mitochondria. Mitochondrial ERα-LBD was also observed by western blot analysis of BC cell fractions confirming the association of ERα-LBD with mitochondria (associated to outer membrane or residing inside mitochondria) (Supplementary Fig. 3a, b).

ERα-LBD expression and localization were further supported by IHC analysis of a human breast cancer tissue microarray (TMA) which included primarily TNBC. To perform this work, we utilized two different antibodies: one targeting the N-terminus of ERα protein which is commonly used in the clinical setting and one targeting the C-terminus, which would detect both ERα-FL and ERα-LBD. We observed that cytosolic staining was much more apparent using the C-terminal antibody (43.7% of total cores; 36.8% of ERα nuclear null/low cores) as compared to the N-terminal antibody (9.4% of total cores; 2.1% of ERα nuclear null/low cores). Notably, levels of cytoplasmic staining were weak compared to nuclear staining of ERα(+) cores and heterogeneous in distribution (Supplementary Fig. 3c–e and Supplementary Data 3). This expression pattern is consistent with our observations by western blot and RNA analyses in ERα(−) models (Figs. 1 and 2).

Given the relative absence of ERα-LBD nuclear localization, we examined its role in canonical ERα signaling, and we observed none of the transcriptional activation functions of full-length ERα. For instance, overexpression of ERα-LBD had no effect on Estrogen Response Element (ERE) luciferase expression or ERα target gene transcription in both ERα-FL(+) and TNBC cells (Supplementary Fig. 3f–k)^35^.

**Fig. 4:**
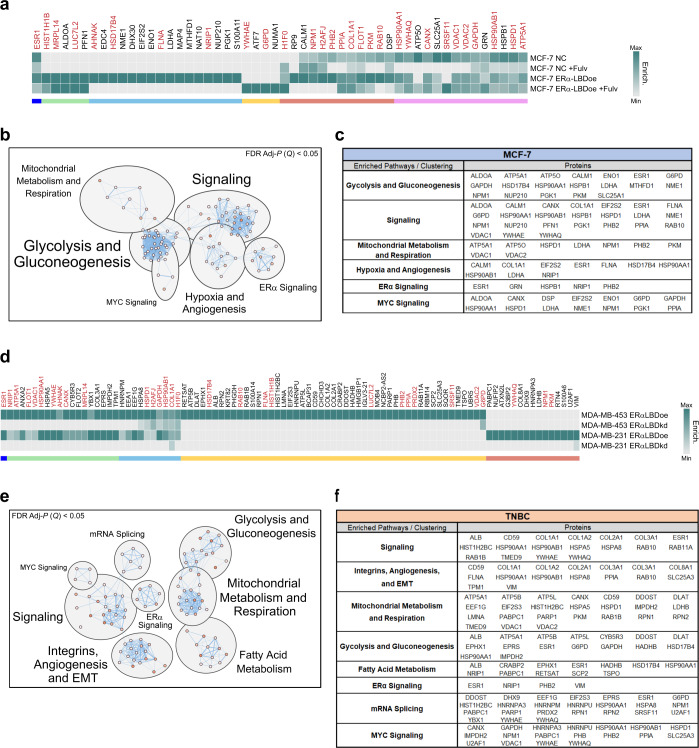
Predicted ERα-LBD network: protein–protein interactions (PPIs) and biological pathways. **a** Heat map showing the enrichment level of proteins that preferentially bind ERα-LBD, based on ERα co-immunoprecipitation (Co-IP) experiments followed by LC/MS analysis. Samples: NC and NC + Fulv (negative controls); ERα-LBDoe, ERα-LBDoe +Fulv (overexpression). Fulv = 1 µM fulvestrant treatment, 24 h. Different comparative criteria between samples were used to identify groups of ERα-LBD protein interactors, color-coded as follows: ERα (blue); proteins present only in ERα-LBD samples (green, cyan and yellow); proteins enriched more in ERα-LBD samples compared to NC, in the absence or presence of fulvestrant treatment (red and purple, respectively). Protein names labeled in dark red color are shared between MCF-7 and TNBC models (see below). **b** ‘Enrichr’ platform based on multiple libraries was used to discover cell pathways in which ERα-LBD PPIs are significantly involved (Adj-*P* (*Q*) < 0.05, FDR test), then clustered and annotated by using Cytoscape software and MCL algorithm. **c** ERα-LBD PPIs contribution to clustered pathways. **d** Same as in **a**. Samples: MDA-MB-453/-231 ERα-LBDoe (overexpression); MDA-MB-453/-231 ERα-LBDkd (knockdown). ERα PPIs and protein groups, color-coded as follow: ERα (blue); proteins enriched in both TNBC cell lines overexpressing ERα-LBD, compared to ERα-LBD silenced cells (green and cyan); enriched in MDA-MB-453 ERα-LBDoe cells only (yellow); enriched in MDA-MB-231 ERα-LBDoe cells only (red). **e**, **f** Same as in **b**, **c**. Source data are provided as Supplementary Data 4 file.

To further establish the functional significance of cytoplasmic and mitochondrial ERα-LBD, we performed co-IP and mass spectrometry experiments aimed at identifying ERα-LBD protein–protein interactions (PPIs). We designed the experiment comparing MCF-7 cells overexpressing ERα-LBD with parental (NC), in the absence or presence of fulvestrant and we identified a total of 52 peptides associated with ERα-LBD (Fig. 4a). Pathway enrichment and neural networking analyses performed on this protein set identified processes involved in carbohydrate metabolism (glycolysis and gluconeogenesis), cell signaling (MYC, mTOR/mTORC1, PIK3C1/AKT, ERα), hypoxia and angiogenesis (HIF1α and VEGFR signaling) and mitochondrial metabolism (oxphos and respiration) (Fig. 4b, c; Supplementary Data 4 and Supplementary Fig. 4a). Comparable experiments in TNBC models overexpressing or knocking down ERα-LBD identified a total of 93 peptides involved in ERα-LBD PPIs, and 29 of them were shared with those found in MCF-7 cells (Fig. 4d). Similarly to the MCF-7 model, enriched pathways included signaling, carbohydrate and mitochondrial metabolism (including also fatty acid β-oxidation) and angiogenesis (Fig. 4e, f; Supplementary Data 4 and Supplementary Fig. 4b).

### ERα-LBD regulates cell metabolism

**Fig. 5:**
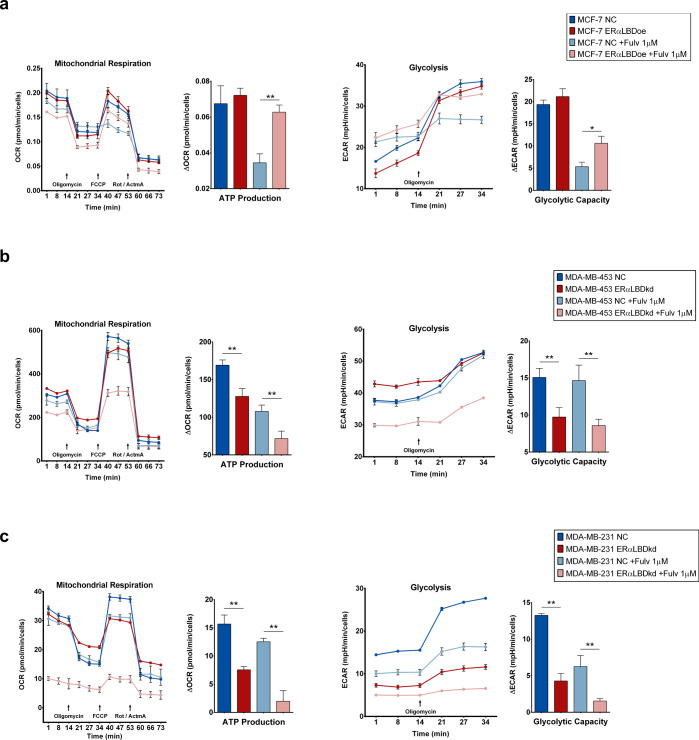
Effect of ERα-LBD ‘gain/loss-of-function’ on BC cell metabolism. **a** Mitochondrial respiration and glycolysis levels in MCF-7 cell clones. Fulvestrant 1 µM (24 h pre-treatment) or vehicle (DMSO) was added to cells. ERα-LBD overexpressing (ERα-LBDoe; in dark/light red) cells were compared to controls (NC; in dark/light blue). Analyses were carried out using XF Cell Mito Stress Kit (Agilent). Respiration was evaluated as oxygen consumption rate (OCR, pmol/min), normalized on cell number. Glycolysis was evaluated as extracellular acidification rate (ECAR, mpH/min), normalized on cell number. The calculation of metabolic parameters was based on ΔOCR and ΔECAR values, following manufacturer’s guidelines. **b**, **c** Mitochondrial respiration and glycolysis levels in MDA-MB-453 and MDA-MB-231 cells. Treatments as in (**a**). Cells with ERα-LBD knockdown (kd; in dark/light red) were compared to controls (NC; in dark/light blue). Analyses were carried out as described above. FCCP p-trifluoromethoxy-phenylhydrazone, Rot rotenone, AtmA antimycin A. Data in the figures are presented as mean ± s.e.m. (*n* = 2 independent experiments). *P < 0.05, **P < 0.01; unpaired *t* test, one-sided. Source data are provided as a Source Data file.

The localization and protein–protein interaction analyses point to a potential metabolic function for ERα-LBD. We speculated ERα-LBD may play a role in regulating glycolysis and mitochondrial respiration, and so assessed the effect of ERα-LBD overexpression or knockdown on key metabolic parameters. In ERα-FL expressing MCF-7, control (NC) and ERα-LBDoe cells showed similar OCR (oxygen consumption rate, index for respiration) and ECAR (extracellular acidification rate, index for glycolysis) under basal conditions. However, following fulvestrant-mediated depletion of ERα-FL and stabilization of ERα-LBD, we observed significantly higher respiratory parameters (ATP production, basal and maximal respiration, and spare respiratory capacity) and higher glycolytic parameters (glycolytic capacity and glycolytic reserve) (Fig. 5a and Supplementary Fig. 5a). In the TNBC models featuring knockdown of ERα-LBD, ERα-LBDkd cells were characterized by reduced levels of respiratory parameters (ATP production, basal and maximal respiration, spare respiratory capacity) and glycolysis (glycolytic capacity and reserve; basal glycolysis only in MDA-MB-231), compared to controls (NC) (Fig. 5b, c and Supplementary Fig. 5b, c). In TNBC models, OCR and ECAR were also tested in the presence of fulvestrant treatment and, despite the overall reduction of respiration (MDA-MB-453 and −231) and glycolysis (MDA-MD-231), metabolic parameters were significantly reduced in ERα-LBDkd cells (Fig. 5b, c and Supplementary Fig. 5b, c).

### ERα-LBD promotes growth and endocrine resistance, in vitro and in vivo

**Fig. 6:**
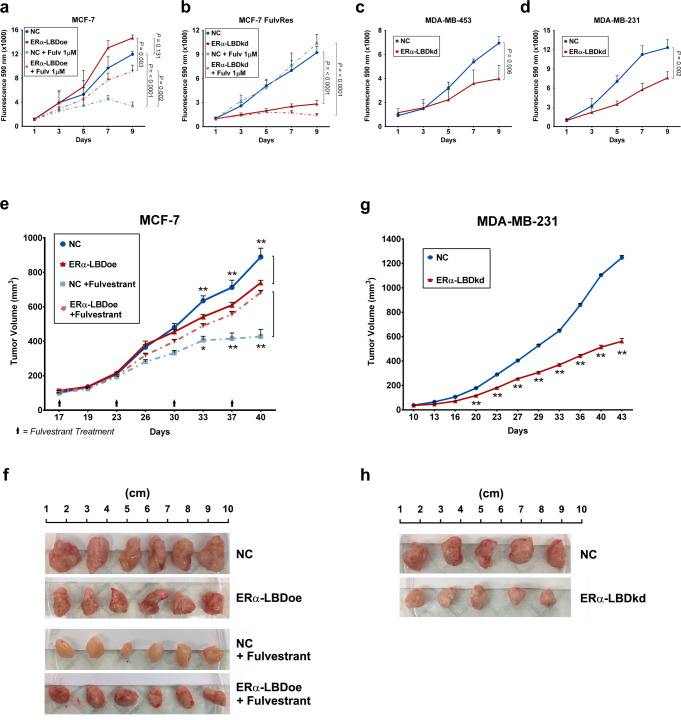
ERα-LBD promotes in vitro and in vivo growth and fulvestrant resistance. **a**–**d** In vitro proliferation of BC cell clones, using resazurin reagent and expressed as fluorescence intensity (absorbance at 590 nm). ERα-LBD overexpression (**a**) or knockdown (**b**–**d**) was compared to controls (NC). In **a** and **b**, cell proliferation was also tested in the presence or absence of fulvestrant 1 µM treatment (*n* = 3 independent experiments). All *P* values describing statistical differences between samples are shown. **e**–**h** Xenografts of BC cells implanted into the mammary fat pads of NOD scid gamma (NSG) mice. Plots in **e** and **f** show mean tumor volumes (mm^3^) as a function of time (days). MCF-7 model: when tumors reached 100 mm^3^, mice were randomized (*n* = 6/group) to weekly treatments with 200 mg/kg fulvestrant injected intramuscularly, and compared to control groups (*n* = 6/group). MDA-MB-231 model: *n* = 5/group. **f**–**h** Images and size of tumors, isolated from animals after reaching endpoint. In **a** and **e**, NC is in blue and ERα-LBD overexpression in red. In **b**–**d** and **g**, NC is in blue and ERα-LBD knockdown in red. All data in the figure are presented as mean ± s.e.m., ns not significant, *P < 0.05, **P < 0.01, two-way ANOVA. Source data are provided as a Source Data file.

Given the impact of ERα-LBD on breast cancer cell metabolism, we investigated the effects of ERα-LBD on cell proliferation and response to endocrine therapy. In vitro, MCF-7 cells overexpressing ERα-LBD, compared to control cells (NC), showed no significant differences in growth. However, upon treatment with fulvestrant, NC cells were potently growth-inhibited (~80% inhibition, *P* > 0.0001, vs. untreated, two-way ANOVA) while ERα-LBDoe cells were only partially growth-inhibited (~40% inhibition, *P* = 0.003, vs. untreated, two-way ANOVA), establishing a role for ERα-LBD in mediating endocrine resistance (Fig. 6a) that was confirmed also by treating MCF-7 NC and ERα-LBDoe cells with tamoxifen (Supplementary Fig. 6a). Moreover, growth of MCF-7 FulvRes cells was significantly impaired after ERα-LDB knockdown (especially under fulvestrant treatment; *P* > 0.0001, two-way ANOVA) whereas no growth inhibition was observed in parental MCF7 FulvRes cells treated with fulvestrant, compared to untreated cells (Fig. 6b). Beyond the effects in ERα-FL(+) cells, we also assessed the effects of ERα-LBD on the growth of TNBC cells. In both MDA-MB-453 and MDA-MB-231 we observed ERα-LBD knockdown to reduce cell proliferation (~50%) (Fig. 6c, d).

To further verify the biologic significance of these effects in vivo, these models were grown orthotopically as xenograft models. As observed in vitro, MCF-7 ERα-LBDoe tumors continued to grow upon fulvestrant treatment while tumors from NC cells were expectedly growth inhibited with fulvestrant (Fig. 6e, f). Moreover, in vivo tumor growth for MDA-MB-231 ERα-LBDkd mice group was markedly reduced, when compared to NC group (Fig. 6g, h). MCF-7 ERα-LBDoe cells showed a growth advantage when cultured in low-attachment (3-D growth) either in the presence or absence of fulvestrant treatment (Supplementary Fig. 6b), whereas ERα-LBD knockdown led to reduced 3-D growth in both MDA-MB-453 and −231 cells (Supplementary Fig. 6c). Similarly, the migratory capacity of BC models was enhanced in ER LBD overexpression models and impaired in knockdown models (Supplementary Fig. 6d, e).

### ERα-LBD expression is associated with proliferation, endocrine resistance, stemness, and metabolism of breast cancer cells

**Fig. 7:**
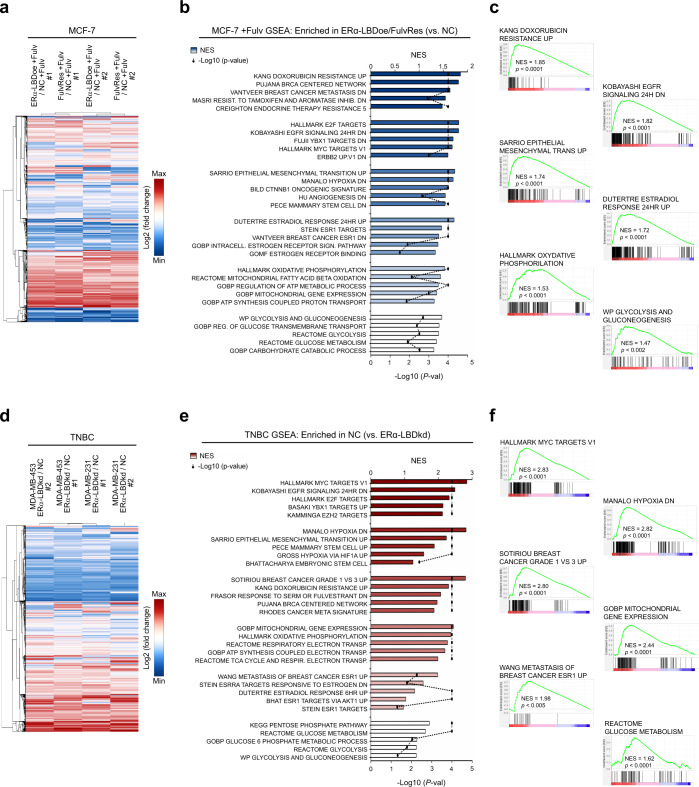
ERα-LBD expression is associated with proliferation, endocrine resistance, stemness, and metabolism of breast cancer cells. Two different BC cell models were used to investigate cell phenotypes associated with ERα-LBD ‘gain/loss-of-function’. **a**–**c** MCF-7 model, with 24 h fulvestrant 1 µM treatment (+Fulv): MCF-7 ERα-LBDoe, MCF-7 FulvRes and MCF-7 NC (control). **d**–**f** TNBC model: MDA-MB-453/-231 ERα-LBDkd (knockdown) and MDA-MB-453/-231 NC (controls). RNA samples were extracted from each cell line and analyzed by RNA-Seq (*n* = 3/sample). **a** Heat map showing differentially expressed genes in MCF-7 ERα-LBDoe (+Fulv) and MCF-7 FulvRes (+Fulv), compared to MCF-7 NC. Gene expression (counts) was determined by using HTSeq, and differential expression was calculated as log_2_(fold change). Only genes with >1 fold change were taken under consideration for the analysis. Hierarchical clustering based on Euclidean Distance was carried out. **b** GSEA analysis based on gene counts and showing gene sets that are both highly and significantly (*P* < 0.05, FDR test) enriched in MCF-7 ERα-LBDoe (+Fulv) and MCF-7 FulvRes (+Fulv), compared to controls. NES normalized enrichment score. Enriched gene sets were grouped and color-coded considering their association with specific gene set collections. Please refer to the “Methods” section for details. **c** Enrichment plots of interest, derived from GSEA analysis described in **b**. **d** Heat map showing differentially expressed genes in TNBC ERα-LBDkd cells, compared to controls (NC). Calculation and analysis was carried out as described in **a**. **e** GSEA analysis showing gene sets that are both highly and significantly (*P* < 0.05, FDR test) enriched in TNBC NC samples compared to TNBC ERα-LBDkd, grouped/color-coded as in **b**. **f** Enrichment plots of interest, derived from GSEA analysis described in **e**. Source data are provided as Supplementary Data 5 file.

Given the growth advantage, drug resistance, and metabolic phenotypes afforded by ERα-LBD, we further evaluated its impact by gene expression analysis. We considered fulvestrant treated MCF-7 ERα-LBDoe and MCF-7 FulvRes cells as ‘gain-of-function’ models compared to MCF-7 control cells (NC). RNA-seq analysis led to the identification of ~17 K differentially regulated genes (Fig. 7a and Supplementary Data 5). Gene set enrichment analysis (GSEA) was performed on these same samples using specific gene set collections related to breast cancer, cell proliferation, and cell metabolism (Supplementary Data 6). Interestingly, gene sets involving cell signaling, breast cancer malignancy/endocrine resistance, EMT and stemness, carbohydrate and mitochondrial metabolism were found to be enriched in the ‘gain of function’ models (Fig. 7b, c and Supplementary Data 5). Conversely, RNA-seq analysis of MDA-MB-453/-231 ERα-LBDkd “loss of function” models identified ~16 K differentially regulated genes in TNBC ERα-LBDkd cells compared to NC cells (Fig. 7d and Supplementary Data 5). Similar to the MCF-7 model, gene sets associated with cell proliferation and survival, cell stemness and metabolism were found to be enriched in TNBC NC cells compared to TNBC ERα-LBDkd (Fig. 7e, f and Supplementary Data 5).

We further explored the role of ERα-LBD in breast cancer stemness and endocrine resistance. Cells from MCF-7 and MCF-7 FulvRes xenografts tumors were screened by flow cytometry and qPCR to measure the expression of CD44, SOX2, and SOX9, markers associated with stemness and endocrine resistance^36–39^. MCF-7 FulvRes tumors were characterized by a higher percentage of CD44^High^ cells which showed higher expression of ESR1-LBD, SOX2 and SOX9, suggesting a correlation between ERα-LBD expression and stemness (Supplementary Fig. 7a, b).

## Discussion

Fulvestrant-resistant breast cancer is frequently observed in the metastatic setting however little data exists on the molecular events underlying resistance^16,24,40–42^. Our analysis of fulvestrant resistant BC models led to the identification of an ERα protein isoform (ERα-LBD, 37.3 kDa MW) lacking the N-terminal transcriptional activation (AF1) and DNA binding domains (DBD) but including a portion of the hinge domain followed by the C-terminal domains LBD (Ligand Binding Domain) and AF2. Not only was ERα-LBD identified in fulvestrant resistant cell lines but also in TNBC models. Unlike full-length ERα which is degraded by fulvestrant, ERα-LBD is stabilized by this agent along with other ERα ligands such as estradiol and tamoxifen, leading to increased ERα-LBD expression (Fig. 1). We hypothesize that the lack of fulvestrant-mediated degradation of ERα-LBD is likely dependent on its different protein sequence/structure, compared to ERα-FL. In fact, many protein elements involved in the regulation of the ubiquitin/proteasome system are missing in ERα-LBD or might be differently exposed/accessible^43^. We also hypothesize that low expression of ERα-LBD in the absence of high levels of ligands may explain why this isoform has not been previously observed in BC cells by others. We also suggest that this feature should be exploited to better understand ERα protein stability and discover novel pharmacologic inhibitors of ERα and its relevant isoforms. Interestingly, a 37 kDa ER species expressed at low levels was previously isolated from the mouse uterus. In accordance to our finding, this ER isoform appeared to have the ligand-binding region and a portion of the hinge domain, but lacked a DNA-binding region^44^.

The identity of ESR1-LBD transcript was determined by analyzing available sequence databases (specifically, FANTOM CAT) and by RNA-seq experiments (Fig. 2). We suggest that the ESR1-LBD transcript ranges from *ESR1* exons E4 to E8 and might utilize an alternative transcription start site and 5′UTR rather than processed by alternative splicing of the full-length transcript (ESR1-FL). The hypothesis of a new putative *ESR1* promoter was supported by preliminary data, describing potential transcriptional activity in the upstream genomic region between E3 and E4 (exon E3a). In addition, we noted reduced expression of the 3′UTR sequence of ESR1-LBD in the ERα(−)/TNBC models, a phenomenon that could alter mechanisms of post-transcriptional inhibition mediated for example by microRNAs. Indeed, we demonstrated that ESR1-LBD is more stable than ESR1-FL and this may explain how a relatively low abundant transcript is translated into a detectable protein (Fig. 2). ESR1-LBD variant has not been previously described and we suggest that the shortening of the 3′UTR might account for this, as it might limit its detection in experiments based on poly(A)-enriched RNA samples.

We demonstrated the presence of the ESR1-LBD transcript in primary BC specimens, examining publicly available RNA-Seq data^32,33^. ESR1-LBD was absent or poorly expressed in non-malignant breast tissue but found to variable levels in ERα(+) tumors, TNBC, and metaplastic breast cancers (MpBC). Remarkably, high levels of ESR1-LBD were identified in 16/17 of metaplastic breast cancers which is one of the most aggressive and chemo-resistant subtypes (Fig. 2). A limitation of this study is the lack of ERα-LBD protein data and thus we cannot ascertain its expression and prevalence in these patient samples. Moreover, data from BC cases with acquired fulvestrant resistance were not available but we hypothesize that expression of ESR1/ERα-LBD would be found in a subset of these cancers. Nevertheless, IHC analyses on a breast cancer tissue microarray allowed us to detect cytoplasmic ERα protein primarily in those specimens lacking nuclear ERα expression, by using an ERα C-terminal antibody (36.8% of samples). Conversely, using an N-terminal antibody (that is typically used to assess the expression of ERα in the clinical setting and which cannot detect ERα-LBD), we identified cytoplasmic staining in only 2.1% of samples. These data support our findings on a truncated ERα isoform expression in BC cells and may account for its lack of detection until now.

The putative function of ERα-LBD was first investigated by examining its localization demonstrating its absence from the nucleus and enrichment in the cytoplasm and mitochondria of breast cancer cells (Fig. 3). We examined ERα-LBD protein–protein interactions by co-IP/MS using overexpression and knockdown models and determined an involvement with glycolysis/gluconeogenesis, mitochondrial metabolism, signaling and angiogenesis (Fig. 4). These observations were further supported by functional analyses revealing metabolic and cell/tumor growth advantages associated with ERα-LBD (Figs. 5 and 6). Moreover, RNA-seq analyses confirmed the association of ERα-LBD expression with gene signatures related to breast cancer and signaling, drug resistance and cell metabolism, stemness, and adaptation to hypoxia (Fig. 7).

An extra-nuclear role for ERα isoforms has been well established most notably by their presence in the plasma membrane and their association with receptor tyrosine kinases and MAPK^45^. However, our data would suggest a distinct role for ERα-LBD as no apparent association with RTKs nor MAPK/ERK was identified. The role of mitochondrial ERα has also been described functioning as a transcription factor interacting with mtDNA and preventing UV-induced apoptosis as well as directly interacting with the mitochondrial protein HADHB^4,46^. Although ERα-LBD has no DNA binding capacity, we also found HADHB among its protein partners, suggesting both unique and overlapping functions for ERα and ERα-LBD in the mitochondria (Fig. 4).

In addition, a role of ERα-LBD in breast cancer stemness and malignancy was suggested by RNA-seq analysis and qPCR demonstrating an enrichment of the ESR1-LBD transcript in fulvestrant resistant CD44^High^ tumor cells (Supplementary Fig. 7). Importantly, ERα-LBD’s role in fulvestrant resistance was also demonstrated by overexpression studies on BC cell growth (Fig. 6) and supported by RNA-seq analysis (Fig. 7). Given the role of stemness in endocrine resistance, we suggest that ERα-LBD may provide a link between these phenomena^15–17,47–49^. Furthermore, the higher abundance of this transcript and protein in cancer stem cells may also explain its relatively low detection in bulk tumor and cell line analyses.

Notably, some of ERα-LBD features are shared with ERα isoform, ERα-36^30,31,50,51^. No cross-detection between ERα-LBD and ERα-36 was found in our models. Specifically, the C-terminal antibody we used for IF, IHC, and co-IP studies does not recognize ERα-36. In addition, the use of primers and an antibody specific for ERα −36 did not lead to its detection in our hands (Supplementary Fig. 2). We suggest that isoforms of ERα may play unique and/or overlapping critical roles in endocrine-resistant disease, TNBC and prognosis. Identifying the unique contributions of these isoforms in breast cancer will be of interest.

In summary, we have identified an ERα isoform “ERα-LBD” in ERα(+) fulvestrant resistant cancer cells, TNBCs and in a subset of human BC specimens. The development of specific strategies for ERα-LBD detection (*e.g*. antibodies, probes) is essential. Further studies are ongoing, to unravel a broader presence and the unique role that ERα-LBD plays in tumorigenesis and cancer progression.

## Methods

### Cell culture and reagents

All cell lines were obtained from ATCC (American Type Culture Collection) and authenticated using short tandem repeat (STR) analysis. Cells were cultured in MEM or RPMI-1640 medium (MSKCC Media Prep, USA) supplemented with 10% heat-inactivated fetal bovine serum (Gibco, USA) and 1% penicillin/streptomycin (Gibco, USA) and maintained at 37 °C and 5% CO_2_ in humidified atmosphere. Reagents used for cell culture and treatments: fulvestrant (Selleckchem, USA), SNX-2112 (Selleckchem, USA), charcoal-stripped fetal bovine serum (CSS, Gibco, USA), 17β-estradiol (E2, Sigma-Aldrich, USA), 4-Hydroxytamoxifen (4-OHT, Sigma-Aldrich, USA).

### Cell proliferation and manipulation

Cells (1000/well) were seeded into 96-well plates and, accordingly to experimental design, vehicle (DMSO 0.01%), fulvestrant 1 µM or tamoxifen 1 µM was added to the medium. Media and drug were replaced every 3 days. At different time points (day 1, 3, 5, 7, and 9), 20 µl of Resazurin (R&D Systems, USA) was added to 180 µl of medium in each well and incubated for 4 h. Fluorescence (absorbance 590 nm) was measured for each well using a SpectraMax M5 microplate reader (Molecular Devices, USA) and correlated to cell growth. To generate fulvestrant-resistant MCF-7 (MCF-7 FulvRes) and T-47D (T-47D FulvRes) cells, cells were cultured in the presence of increasing concentrations of fulvestrant (0.1–1 µM). Cells were deemed resistant when able to actively proliferate in 1 µM fulvestrant. MCF-7 Y537S CRISPR knock-in cells were generated as described elsewhere^24^. PDX models of ERα(+)/(−) metastatic BC were generated and maintained as described elsewhere^17^. BC cell clones overexpressing ERα-LBD (ERα-LBDoe) were generated using stable retroviral transduction. Briefly, ERα-LBD coding sequence was cloned into pBABE-Puro vector (Addgene, USA; Supplementary Data 7). To generate retroviral particles, three plasmids were co-transfected into 293T packaging cells using Lipofectamine 2000 (Invitrogen, USA): pBABE-Puro-ERαLBDoe, pCMV-VSV-G and pUMVC (Addgene, USA). Viral supernatants were collected 48 h and 72 h later, centrifuged to remove cell debris, filtered through 0.45-µm filters (Millipore, USA) then used with polybrene 8 µg/ml polybrene (Santa Cruz Biotechnology, USA) to transduce BC cell lines. After 2 days, stable clones were selected with puromycin 2 µg/ml. BC cell clones with ERα-LBD knockdown (ERα-LBDkd) were generated using stable lentiviral transduction and CRISPR/CAS9 technology for genome editing. Briefly, three different guide RNA sequences targeting ESR1 gene (exon #6) were cloned in the BsmBI site of lentiCRISPRv2 vector (Addgene, USA; Supplementary Data 7) to create a pool of pCR-ERαLBDkd lentivectors. To generate lentiviral particles, the following plasmids were co-transfected into 293T packaging cells: pCR-ERαLBDkd (pool), pCMV-VSV-G and psPAX2 (Addgene, USA). Infection and selection procedures: same as retroviral approach (see above).

### Western blot

Cells were homogenized in RIPA lysis buffer for protein extraction, supplemented with protease inhibitors (Thermo Scientific, USA). Denatured proteins were separated in 12% SDS–PAGE and then transferred onto nitrocellulose papers (Pall, USA). After blotting, nitrocellulose papers were incubated with specific antibodies. Primary antibodies: anti-ERα antibody D8H8, 1:2000 (Cell Signaling Technology, USA, #8644); anti-ERα F-10, 1:1000 (Santa Cruz Biotechnology, USA, #sc-8002), anti-ERα 1D5, 1:1000 (Invitrogen, USA, #MA5-13191), anti-ERα-36, 1:200 (Alpha Diagnostic International, USA, #ERA361-A); anti-β-actin 13E5, 1:2000 (Cell Signaling Technology, USA, #8457); anti-HDAC2, 1:1000 (Cell Signaling Technology, USA, #2540); anti-VDAC1 N-18 (Santa Cruz Biotechnology, USA, #sc-8828). Secondary antibodies (HRP conjugated): anti-rabbit, 1:5000 (Cell Signaling Technology, USA, #7074); anti-mouse, 1:2000 (Cytiva, USA, #RPN4201). Immunolabelling was visualized using ECL procedure (PerkinElmer, USA). Uncropped and unprocessed scans of the most important blots are supplied in the Source Data file. All blots derive from the same experiment and they were processed in parallel.

### Gel extraction, mass spectrometry, and protein structure analysis

Protein lysates from MCF-7 FR and MDA-MB-231 (both treated with fulvestrant 1 µM for 24 h) were obtained using IP buffer (NaCl 150 mM, EDTA 0.5 mM, NP-40 0.5% (v/v), Tris-HCl 10 mM pH 7.4, PMSF 1 mM) and immunoprecipitated using anti-ERα antibody (1:100, Cell Signaling Technology, USA, #8644). IP samples were run on two electrophoresis gels, one used for staining with SimplyBlue Safe Stain (Invitrogen, USA) and band extraction, the other for WB sample check. In-gel digestion was performed using the method by Shevchenko et al.^52^. Briefly, gel bands were excised, washed with acetonitrile and 100 mM ammonium bicarbonate solution (1:1) for 30 min, dehydrated with 100% acetonitrile for 10 min and dried in a speed-vac for 10 min without heat. Gel slices were reduced with 5 mM DTT for 30 min at 56 °C in a thermo-mixer with gentle mixing, removed, allowed to cool to room temperature then alkylated with 11 mM IAA for 30 min in the dark. Gel slices were washed with 100 mM ammonium bicarbonate and 100% acetonitrile for 10 min each. Excess acetonitrile was removed and the slices dried in a speed-vac for 10 min without heating. Gel slices were then rehydrated in a solution of 25 ng/ml trypsin in 50 mM ammonium bicarbonate on ice for 30 min. Digestions were performed overnight at 37 °C in a thermo-mixer with gentle mixing. Digested peptides were collected and further extracted from gel slices in extraction buffer (5% formic acid and 50% acetonitrile, 1:2 vol/vol) at high-speed mixing. Extractions were combined and dried down in a vacuum centrifuge. Peptides were desalted with C18 resin-packed stage-tips, lyophilized to dryness, then re-constituted in 3% acetonitrile/0.1% formic acid for LC-MS/MS analysis. LC-MS/MS Analysis LC-MS/MS was performed using a Waters NanoAcquity LC system (with a 100 mm inner diameter × 10 cm length C18 column (1.7 mm BEH130; Waters, USA) configured with a 180 mm × 2 cm trap column coupled to a Thermo Q-Exactive Plus orbitrap mass spectrometer (Thermo Scientific, USA). Trapping was performed at 15 ml/min 0.1% formic acid (Buffer A) for 1 min. The LC gradient was 0.5 to 50% B (100% acetonitrile; 0.1% formic acid) over 90 min at 300 nl/min. MS data were collected in data-dependent acquisition (DDA) mode utilizing a top ten precursor ion selection for HCD fragmentation. Full MS scans were performed with the following parameters: Resolution: 70,000; AGC target: 1e6; Maximum IT: 50 ms; Scan Range: 400 to 1600 m/z. DDA parameters were as follows: Resolution: 17,500; AGC target 5e4; Maximum IT: 50 ms; Isolation window: 1.5 *m/z*; NCE: 27; Minimum AGC target: 2e3; Intensity Threshold: 4e4; Dynamic Exclusion: 15 s; Charge exclusion: unassigned, 1, 6–8, >8. All MS/MS samples were analyzed using Mascot (Matrix Science, UK). Mascot was set up to search the SwissProt_sprot_20170705_20180523 database (selected for Homo sapiens, unknown version, 20215 entries) assuming the digestion enzyme trypsin. Mascot was searched with a fragment ion mass tolerance of 0.080 Da and a parent ion tolerance of 10.0 PPM. Carbamidomethyl of cysteine was specified in Mascot as a fixed modification. Deamidated of asparagine and glutamine, oxidation of methionine, acetyl of the N-terminus and phosphorylation of serine, threonine and tyrosine were specified in Mascot as variable modifications. Mass spectrometry data were further processed using Scaffold software (Proteome Software Inc., USA). Peptide identifications were accepted if they could be established at >90.0% probability to achieve an FDR <1.0% by the Peptide Prophet algorithm with Scaffold delta-mass correction. Protein identifications were accepted if they could be established at >89.0% probability to achieve an FDR <1.0% and contained at least 1 identified peptide. Protein probabilities were assigned by the Protein Prophet algorithm. Proteins that contained similar peptides and could not be differentiated based on MS/MS analysis alone were grouped to satisfy the principles of parsimony. Proteins sharing significant peptide evidence were grouped into clusters. Protein sequence and features were analyzed using Sequence Analysis software (Informagen, USA). Three-dimensional protein models were generated by Phyre^2^ web portal (www.sbg.bio.ic.ac.uk)^53^.

### ESR1-LBD transcript variant prediction

Comprehensive ESR1 transcript variants annotation was obtained from Ensembl Genome Browser (www.ensembl.org). We used ZEMBU Genome Browser (https://fantom.gsc.riken.jp/zenbu/) for variant prediction study, showing data collected on ESR1 gene, based on FANTOM5 and FANTOM CAT analyses^27,28^. For ESR1 gene and transcripts details please refer to this link: https://fantom.gsc.riken.jp/zenbu/gLyphs/#config=eljPd0OTPhIoifL81WStGC;loc=hg19::chr6:152118533..152432431+.

### RNA capture-sequencing and ESR1 transcripts analysis

Total RNA was extracted as described elsewhere^54^ and samples (100 ng) were input in the RNA library construction using the KAPA RNA Hyper library prep kit (Roche, Switzerland). Customized adapters with unique molecular indexes (UMI) (Integrated DNA Technologies, USA) and Sample-specific dual-indexes primers (Integrated DNA Technologies, US) were added to each library. Each RNA library was pooled for hybridization capture with customized ESR1 Panel (Integrated DNA Technologies, USA) using a capture protocol modified from NimbleGen SeqCap Target Enrichment system (Roche, Switzerland). Libraries were then sequenced on an Illumina MiniSeq with paired-end reads (×150 cycles, 1.4 millions reads/sample). Raw sequencing data output was processed for expression analysis using STAR Aligner^55^, DEXSeq-count^56^, Cluster 3.0 and Java TreeView^57,58^, DEXSeq and plotDEXSeq^56^, Kallisto^59^ (Supplementary Fig. 8a).

### RNA extraction and real-time qPCR

Total RNA was extracted from cells using Trizol reagent (Invitrogen, USA), according to the manufacturer’s instructions. Extracted RNA samples were quantified and then treated with DNase I to remove any genomic DNA contamination, using Ambion DNase I kit (Invitrogen, USA). Reverse transcription was carried out using iScript™ Select cDNA Synthesis Kit (Bio-Rad, USA). cDNA levels were analyzed by real-time PCR using TaqMan Universal PCR Master Mix or SYBR Select Master Mix reagents and ViiA 7 Real-Time PCR system, according to the manufacturer’s instructions (Applied Biosystems, USA). Melting curve data were collected to check PCR specificity. Samples were run in triplicate and mRNA levels were normalized against those of RPLP0 or β-actin. Relative expressions were calculated using the formula 2^−2ΔCt^ values. Expression data were obtained by using QuantStudio™ Real-Time PCR Software (Applied Biosystem, USA). RT-PCR was carried out by using OneTaq Hot Start 2X Master Mix (New England Biolabs, USA). PCR samples were separated on a 1.5% agarose gel and results visualized using Gel Doc XR + Imaging System (Bio-Rad, USA). For RNA Decay Assay, BC cells were seeded in 6-well plates and treated with actinomycin-D 5 µg/ml (Sigma-Aldrich, USA). RNA samples were collected at different time points (0, 4, 8, 24, 48 h). All PCR primers are summarized in Supplementary Data 7.

### Transfection and luciferase assay

Cells were seeded in 24-well plate (2 × 10^5^ cells/well) 1 day before transfection and treatments were added accordingly to the experimental design (not treated, vehicle/DMSO 0.01% or fulvestrant 1 µM). For the putative promoter experiment, three different human genomic DNA regions representing putative ESR1-LBD promoter (pESR1-LBD-1/2/3) were cloned into pGL3.basic plasmid (Promega, USA; Supplementary Data 7). BC cells were co-transfected with pGL3.basic (NC) or p-ESR1-LBD reporter plasmids (750 ng) and pRL-TK Renilla Luciferase plasmid (75 ng) (Addgene, USA) by using Lipofectamine 2000 (Invitrogen, USA) and following reagent protocol. For ERα-driven transcriptional activity experiment, BC cells were co-transfected with 3x-ERE-TATA-Luciferase reporter plasmid (750 ng) and pRL-TK Renilla Luciferase plasmid (75 ng) (Addgene, USA) by using the same procedure described above. Cells were harvested 24 h after transfection and cell lysates were used for Dual-Luciferase® Reporter Assay System analysis, according to the manufacturer’s instructions (Promega, USA). Luciferase bioluminescence measurements were performed with the Veritas™ Microplate Luminometer (Promega, USA). For each sample, Firefly luciferase activity was normalized against Renilla luciferase activity.

### Breast cancer patients data

RNA-seq FASTQ files of 135 breast cancer were obtained from the European Nucleotide Archive, ENA (Study Accession: PRJNA251383; Supplementary Data 2)^32^. RNA-seq FASTQ files of 17 metaplastic breast cancer were kindly provided by Dr. Jorge S Reis-Filho (Supplementary Data 2)^33^. Raw data were processed by using DEXSeq-count^53^, Kallisto^54^ and Morpheus (https://software.broadinstitute.org/morpheus).

### Immunohistochemistry and confocal microscopy

ERα immunostaining (IHC) was performed on Benchmark Ultra using the ultraView DAB Detection kit (Ventana, USA). Antigen retrieval was performed onboard with UltraCC1 buffer (pH 8.2–8.5) at 95 °C for 52 min. Primary antibody (anti-ERα antibody D8H8, Cell Signaling Technology, USA, #8644) 1:100 for 28 min at 37 °C. Secondary antibody 1:100 for 1 h. Images were obtained using a Zeiss Axiovert Widefield Microscope and Zeiss ZEN software (Carl Zeiss, Germany). Immunofluorescence was performed using Leica Bond RX stainer and the Bond Polymer Refine Detection kit (Leica, Germany). Antibodies and detection: OXPHOS (2 µg/ml, Invitrogen, USA, #A-21347); ERα D8H8 (1 µg/ml, Cell Signaling Technology, USA, #8644); Alexa Fluor Tyramide signal amplification reagents (Life Technologies, USA); 4′, 6-diamidino-2-phenylindole (DAPI, Sigma-Aldrich, USA). Slides were mounted in Mowiol 4–88 (Calbiochem, USA). Confocal imaging was performed on a Leica SP8 inverted microscope (Leica, Germany). Image processing and analysis (2-D and 3-D) was performed using Imaris software (Bitplane, CH).

### Cell fractionation for western blot

Whole-cell lysate. Cells were pelleted and homogenized in lysis buffer (50 mM Tris-HCl, pH 7.5, 2 mM EDTA, 100 mM NaCl, 1% Triton X-100, protease inhibitors). Cell lysates were incubated 1 h on ice and centrifuged at 12,000 g at 4 °C for 20 min to collect supernatants (sample W). Nuclear and cytosolic fractions. Pelleted cells were resuspended in nuclear lysis buffer (10 mM HEPES; pH 7.5, 10 mM KCl, 0.1 mM EDTA, 0.5% Nonidet‐40, 0.5 mM PMSF, protease inhibitors). Cells were allowed to swell on ice for 15‐20 min with intermittent mixing to disrupt cell membranes and then centrifuged at 12,000 × *g* at 4 °C for 10 min. Supernatant were transferred into a new tube and used as cytosolic fraction (sample C). The pelleted nuclei were washed with nuclear lysis buffer and resuspended in nuclear extraction buffer (20 mM HEPES pH 7.5, 400 mM NaCl, 1 mM EDTA, 1 mM DTT, 1 mM PMSF, protease inhibitors) and incubated in ice for 30–60 min. Nuclear extract (supernatant) was collected by centrifugation at 16,000 × *g* for 15 min at 4 °C and transferred into a new tube (sample N). Mitochondrial fraction. Cells were collected by centrifugation at ~500 × *g* for 10 min then resuspended in 10 packed cell volumes of NKM buffer, x2 (Tris-HCl 1 M pH 7.4, NaCl 2 M, KCl 1 M, MgCl_2_ 0.5 M). Cells were pelleted and resuspended in 6 packed cell volumes of homogenization buffer (Tris-HCl 1 M pH 6.7, KCl 1 M, MgCl_2_ 0.5 M PMSF 200 mM). Cell suspension was transferred to a glass homogenizer, incubate for 10 min on ice, then homogenized using a tight pestle. The level of cell breakage was checked under the microscope. Homogenate was poured into a conical centrifuge tube containing 2 packed cell volume of 2 M sucrose solution and mixed gently. Unbroken cells, nuclei and large debris were pelleted at 1200 g for 5 min. The supernatant was transferred to another tube containing 1.5 packed cell volume of 2 M sucrose and mixed gently. Mitochondrial fraction was collected by centrifuging at 12,000 × *g* for 15 min and resuspended in isotonic mitochondrial buffer (10 mM Hepes buffer pH 8.0, 250 mM sucrose, 0.5 mM EGTA). The suspension was split 1:2. One half, centrifuged at 12,000 × *g* for 15 min and resuspended in lysis buffer. After incubation of 30–60 min on ice, mitochondrial fraction (supernatant) was collected by centrifugation at 12,000 × *g* for 15 min (sample M). The other half was incubated with proteinase K (50 µg/ml, Thermo Fisher) for 30 min at 25 °C. PMSF was added to a final concentration of 2 mM to terminate the proteinase K activity, followed by 10 min of incubation. PK-treated mitochondria were collected by centrifugation at 12,000 × *g* for 10 min (sample M*).

### TMA immunohistochemistry

ERα immunostaining (IHC) was performed on Benchmark Ultra using the ultraView DAB Detection kit (Ventana, USA). Antigen retrieval was performed onboard with UltraCC1 buffer (pH 8.2–8.5) at 95 °C for 52 min. The primary anti-ERα antibody (D8H8, Cell Signaling Technology, USA, #8644) was incubated 1:100 for 28 min at 37 °C. Secondary antibody 1:100 for 1 h. Hematoxylin/eosin counterstain was also carried out. Slides were scanned by Leica Aperio AT2 (Leica Biosystems, USA) whole slide scanners. Images from ERα staining by using 6F11 antibody were kindly obtained by US BioMax Inc., USA. For each sample/core, IHC grading was assessed considering the intensity of the staining and the number of stained cells.

### ERα co-immunoprecipitation and LC/MS

Protein were extracted using Co-IP buffer (NaCl 150 mM, EDTA 0.5 mM, NP-40 0.5% (v/v), Tris-HCl 10 mM pH 7.4, PMSF 1 mM). Immunoprecipitation (IP): anti-ERα antibody D8H8 1:75 (Cell Signaling Technology, USA, #8644) and Dynabeads™ Protein A (Invitrogen, USA). IP samples were washed four times with 50 mM ammonium bicarbonate buffer and collected by centrifugation. Samples were then digested overnight with 2 µg trypsin in 80 µl of 50 mM ammonium bicarbonate at 37 °C. Desalted and dried peptides were reconstituted in 10 µl 0.1% (vol/vol) formic acid and analyzed (4 µl) by microcapillary liquid chromatography with tandem mass spectrometry. Pathway enrichment analysis was performed by using Enrichr platform (https://maayanlab.cloud/Enrichr/)^60^. Enriched pathways network, clustering, and protein–protein interaction (PPI) network analyses were carried out using Cytoscape, EnrichmentMap, AutoAnnotate and stringApp softwares^61^.

### Metabolic assay

Mitochondrial respiration and glycolysis were assessed using Seahorse extracellular flux analyzer (XFe96) and Seahorse XF Cell Mito Stress Test Kit (Agilent Technologies, USA). Cells were seeded in 96-well plates (2 × 10^4^ cells/well), with or without Fulvestrant 1 µM 24 h pre-treatment. Raw data output was collected and analyzed using Wave Software (Agilent Technologies). Procedures and OCR/ECAR data interpretation were carried out accordingly to the manufacturer’s guidance.

### Animal models

NOD scid gamma (NSG) female mice at age of 6–8 weeks were obtained from Jackson Laboratory (USA) and maintained in pressurized ventilated caging. To sustain tumor growth in MCF-7 models, 17β-estradiol pellets (0.18 mg) were implanted subcutaneously 3 days before BC cells injection. For both MCF-7 and MDA-MB-231 models, cancer cells were injected in the mammary fat pads (MFPs) of mice. For each experimental sample, cell suspensions were mixed with an equal volume of Matrigel (BD Biosciences, USA). For the MCF-7 model, injectable fulvestrant (Faslodex®, AstraZeneca, UK) was given intramuscularly in the tibialis posterior/popliteal muscles (200 mg/kg injection, once a week) for 20 days. Control mice received isotype control (placebo) or PBS injection. Tumor volumes were measured with vernier calipers starting from 14 days after cell implantation. Animals were sacrificed as they reached an experimental endpoint. All procedures and experiments were completed in accordance with the Guidelines for the Care and Use of Laboratory Animals and were approved by the Institutional Animal Care and Use Committees at MSKCC (MSKCC#12-10-016).

### Microscopy, 3-D growth, and wound healing assay

MCF-7 cells (WT and FulvRes) were seeded into six-well plate (1 × 10^5^ cells/well) and treated accordingly to the experimental design. After controls reached 100% confluence, images of cells were captured with Zeiss Axiovert Microscope and processed with Zeiss ZEN software (Carl Zeiss, Germany). For 3-D growth, BC cell clones were suspended sparsely and plated in 24-well ultra-low attachment plates (Corning, USA), 3 × 10^3^ cells/well. Treatments (DMSO 0.01% or fulvestrant 1 µM) were added to medium accordingly to experimental design. After 5 days, representative images of cells were acquired with a Zeiss Axiovert Microscope. Quantification of cell 3-D proliferation was based on the optical density (OD 600 nm) of cell suspensions and determined using a SpectraMax M5 microplate reader (Molecular Devices, USA). For the wound healing assay, about 1 × 10^5^ cells were seeded into each well of 12-well plates and cultured until 100% confluence. The ‘scratch’ was created with a p200 pipet tip on the cell monolayer through the center of the well. The debris was removed by washing the well with 1 ml of culture medium and then 1 ml of medium (with 1% serum) was added into each well. Treatments (DMSO 0.01% or fulvestrant 1 µM) were also added accordingly to the experimental design. The plate was incubated at 37 °C, and images of the ‘scratches’ were captured at various time points with a Zeiss Axiovert Microscope. The width of scratch was measured with FIJI software.

### RNA sequencing

Total RNA was extracted from cell lines as described elsewhere^54^ and sequenced on an Illumina HiSeq instrument (2 × 150 paired-end, 100 million reads/sample) by GENEWIZ, LLC. (USA). Raw sequencing data output was processed for downstream analyses using the following bioinformatic tools: STAR (reads alignment)^55^, HTSeq (gene expression)^62^, Cluster 3.0 and Java TreeView (hierarchical clustering and heat map)^57,58^, GSEA (enrichment of gene sets)^63^ (Supplementary Fig. 8b and Supplementary Data 6).

### Flow cytometry (FACS)

For FACS/flow analyses, tumors were digested in sterile Epicult media (StemCell Technology, Canada), minced with sterile razor blades and incubated for 3 h in the presence of collagenase/hyaluronidase (1,000 Units/sample). Cells were washed with sterile filtered PBS supplemented with 1% BSA (PBS-BSA 1%) and filtered through a 40 mm nylon mesh (BD Biosciences, USA). Cells were then stained in a volume of 100 µL (PBS-BSA 1%) with CD44-APC antibody 100 ng/10^6^–10^8^ cells (IM7, eBiosciences, USA, # 17-0441-82) on ice for 30 min and analyzed by flow cytometry at the MSKCC’s flow core with BD FACS Aria I instrument (BD Biosciences, USA). Samples were analyzed for cell population distribution and sorted for viability (DAPI^neg^) and CD44 expression. For flow plot analyses, samples were run using FlowJo 7.5 software (Tree Star, USA).

### Statistics

Data were expressed as mean ± s.e.m. Student’s *t* test (unpaired/paired; one/two-tailed) or Analysis of Variance (two-way ANOVA, followed by Fisher’s test or Sidak’s/Tukey’s correction for multiple comparisons) were used to assess the statistical significance of the differences. For DEXSeq analysis, *P* values were adjusted for multiple testing using Benjamini & Hochberg (BH) correction (FDR). For Enrichr analysis, *P* values were computed from the Fisher exact test and FDR adjusted *P* values (*Q* values) were used to filter enriched pathways for Cytoscape and Enrichment Map analysis. For statistics on GSEA analysis (FDR test), please refer to official website (www.gsea-msigdb.org). Differences were considered statistically significant at *P* < 0.01 and *P* < 0.05.

## Supplementary information

Supplementary Information

Supplementary Data 1

Supplementary Data 2

Supplementary Data 3

Supplementary Data 4

Supplementary Data 5

Supplementary Data 6

Supplementary Data 7

## Data Availability

All data supporting the findings of this study are available with the article (including Supplementary information) or from the corresponding author upon reasonable request. The source data underlying Figs. 1a, b, d, 5a–c, 6a–e, g and Supplementary Figs. 1a–c, f–h, 2b, f, g, i, 3a, f–k, 5a–c, 6a–e and 7b are provided as a Source Data file. The source data underlying Figs. 2a–h, 4a–f and 7a–f are provided as Supplementary Data 2, 4, 5, respectively. RNA-seq data have been deposited to ArrayExpress with accession numbers E-MTAB-10733 and E-MTAB-10738. MpBC RNA-seq data have been previously deposited to the Sequence Read Archive (SRP070780)^33^. The mass spectrometry proteomics data have been deposited to the ProteomeXchange Consortium via the PRIDE partner repository with the dataset identifiers PXD027087 and PXD027088.

## Source data

Source Data File
