# Supplementary material for: ERα-LBD, an isoform of estrogen receptor alpha, promotes breast cancer proliferation and endocrine resistance
Source: NPJ Breast Cancer. 2022 Aug 23;8:96. doi: 10.1038/s41523-022-00470-6 (PMC9399095; doi:10.1038/s41523-022-00470-6)
Supplement: Supplementary file 1 — Supplementary Information [file 41523_2022_470_MOESM1_ESM.pdf]

Supplementary Figure 1

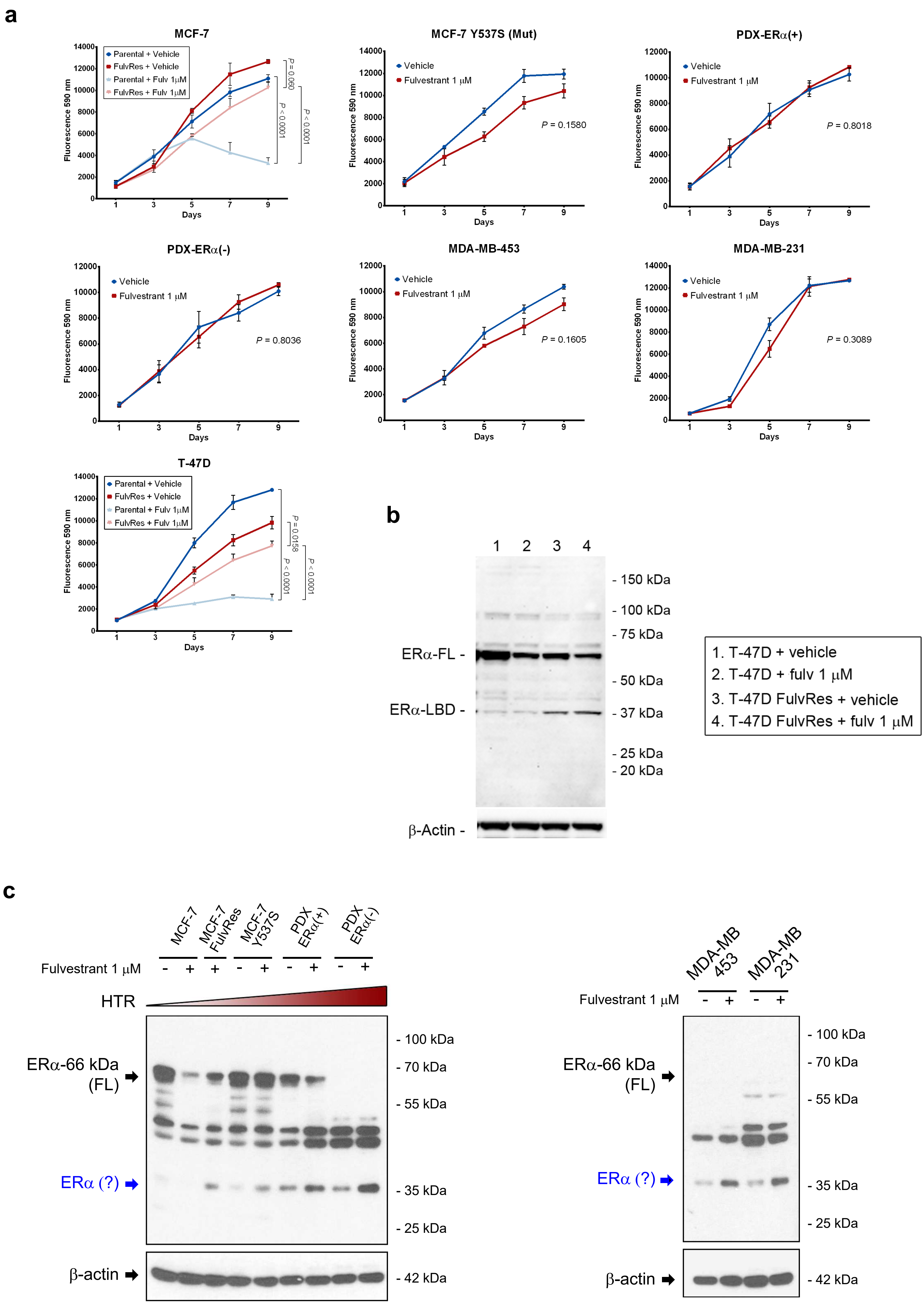

d

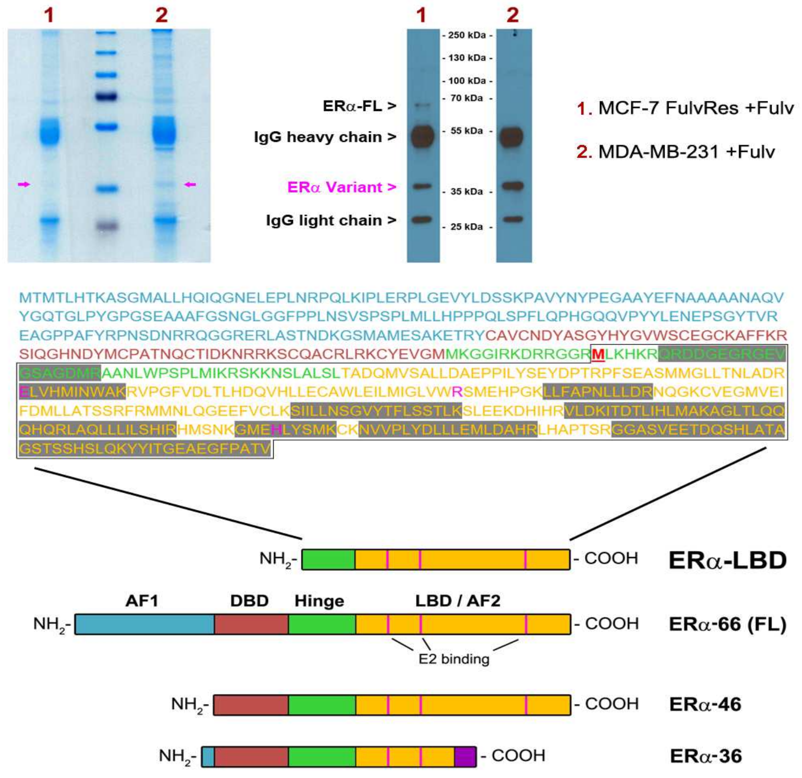
1
2
1
2
- 250 kDa -
- 130 kDa -
- 100 kDa -
- 70 kDa -
- 55 kDa -
- 35 kDa -
- 25 kDa -
ERα-FL >
IgG heavy chain >
ERα Variant >
IgG light chain >
1. MCF-7 FulvRes +Fulv
2. MDA-MB-231 +Fulv
MTMTLHTKASGMALLHQIQGNELEPLNRPQLKIPLERPLGEVYLDSSKPAVYNYPEGAAYEFNAAAAANAQV
YGQTGLPYGPGSEAAAFGSNGLGGFPPLNSVSPSPLMLLHPPPQLSPFLQPHGQQVPYYLENEPSGYTVR
EAGPPAFYRPNSDNRRQGGRERLASTNDKGSMAMESAKETRYCAVCNDYASGYHYGVWSCEGCKAFFKR
SIQGHNDYMCPATNQCTIDKNRRKSCQACRLRKCYEVGMMKGGIRKDRRGGRMLKHKRQRDDGEGRGEV
GSAGDMRAANLWPSPLMIKRSKKNSLALSLTADQMVSALLDAEPPILYSEYDPTRPFSEASMMGLLTNLADR
ELVHMINWAKRVPGFVDLTLHDQVHLLECAWLEILMIGLVWRSMEHPGKLLFAPNLLLDRNQGKCVEGMVEI
FDMLLATSSRFRMMNLQGEEFVCLKSIILLNSGVYTFLSSTLKSLEEKDHIHRVLDKITDTLIHLMAKAGLTLQQ
QHQRLAQLLLILSHIRHMSNKGMEHLYSMKCKNVVPLYDLLLEMLDAHRLHAPTSRGGASVEETDQSHLATA
GSTSSHSLQKYYITGEAEGFPATV
NH2- - COOH ERα-LBD
AF1 DBD Hinge LBD / AF2
NH2- - COOH ERα-66 (FL)
E2 binding
NH2- - COOH ERα-46
NH2- - COOH ERα-36

e

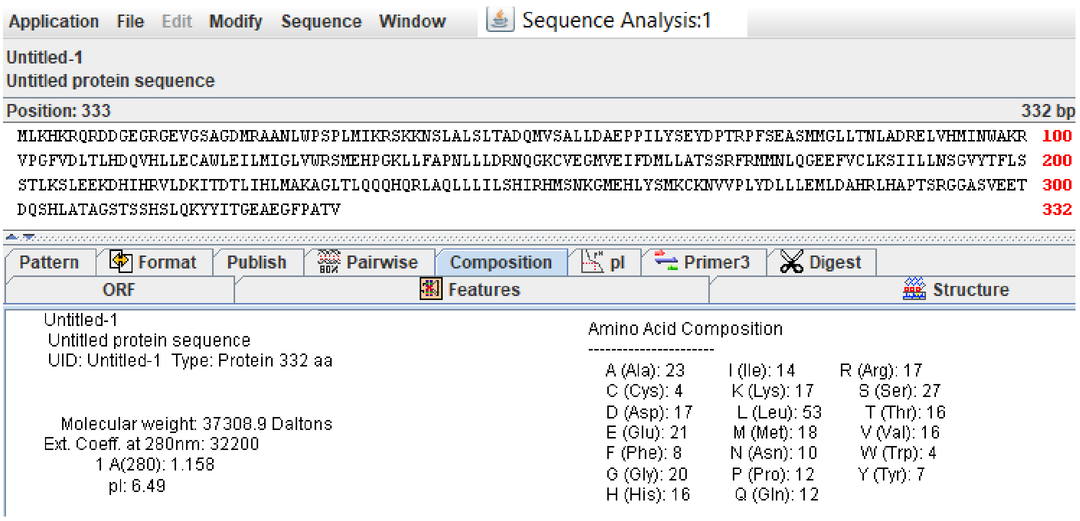
Application File Edit Modify Sequence Window
Sequence Analysis:1
Untitled-1
Untitled protein sequence
Position: 333
332 bp
MLKHKRQRDDGEGRGEVGSAGDMRAANLWPSPLMIKRSKKNSLALSLTADQMVSALLDAEPPILYSEYDPTRPFSEASMMGLLTNLADRELVHMINWAKR 100
VPGFVDLTLHDQVHLLECAWLEILMIGLVWRSMEHPGKLLFAPNLLLDRNQGKCVEGMVEIFDMLLATSSRFRMMNLQGEEFVCLKSIILLNSGVYTFLS 200
STLKSLEEKDHIHRVLDKITDTLIHLMAKAGLTLQQQHQRLAQLLLILSHIRHMSNKGMEHLYSMKCKNVVPLYDLLLEMLDAHRLHAPTSRGGASVEET 300
DQSHLATAGSTSSHSLQKYYITGEAEGFPATV 332
Pattern
Format
Publish
Pairwise
Composition
pI
Primer3
Digest
ORF
Features
Structure
Untitled-1
Untitled protein sequence
UID: Untitled-1 Type: Protein 332 aa
Molecular weight: 37308.9 Daltons
Ext. Coeff. at 280nm: 32200
1 A(280): 1.158
pI: 6.49
Amino Acid Composition
A (Ala): 23
C (Cys): 4
D (Asp): 17
E (Glu): 21
F (Phe): 8
G (Gly): 20
H (His): 16
I (Ile): 14
K (Lys): 17
L (Leu): 53
M (Met): 18
N (Asn): 10
P (Pro): 12
Q (Gln): 12
R (Arg): 17
S (Ser): 27
T (Thr): 16
V (Val): 16
W (Trp): 4
Y (Tyr): 7

f

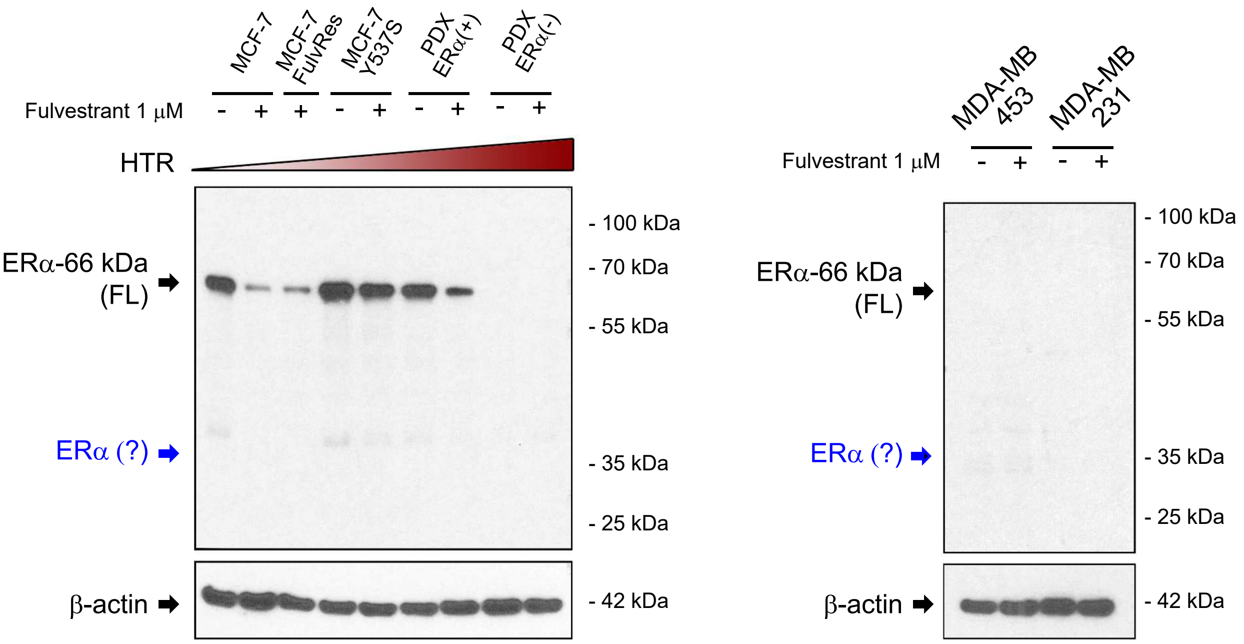
MCF-7
MCF-7 FulvRes
MCF-7 Y537S
PDX ERα(+)
PDX ERα(-)
Fulvestrant 1 μM
- + + - + - + - +
HTR
ERα-66 kDa (FL)
ERα (?)
β-actin
- 100 kDa
- 70 kDa
- 55 kDa
- 35 kDa
- 25 kDa
- 42 kDa
MDA-MB 453
MDA-MB 231
Fulvestrant 1 μM
- + - +
ERα-66 kDa (FL)
ERα (?)
β-actin
- 100 kDa
- 70 kDa
- 55 kDa
- 35 kDa
- 25 kDa
- 42 kDa

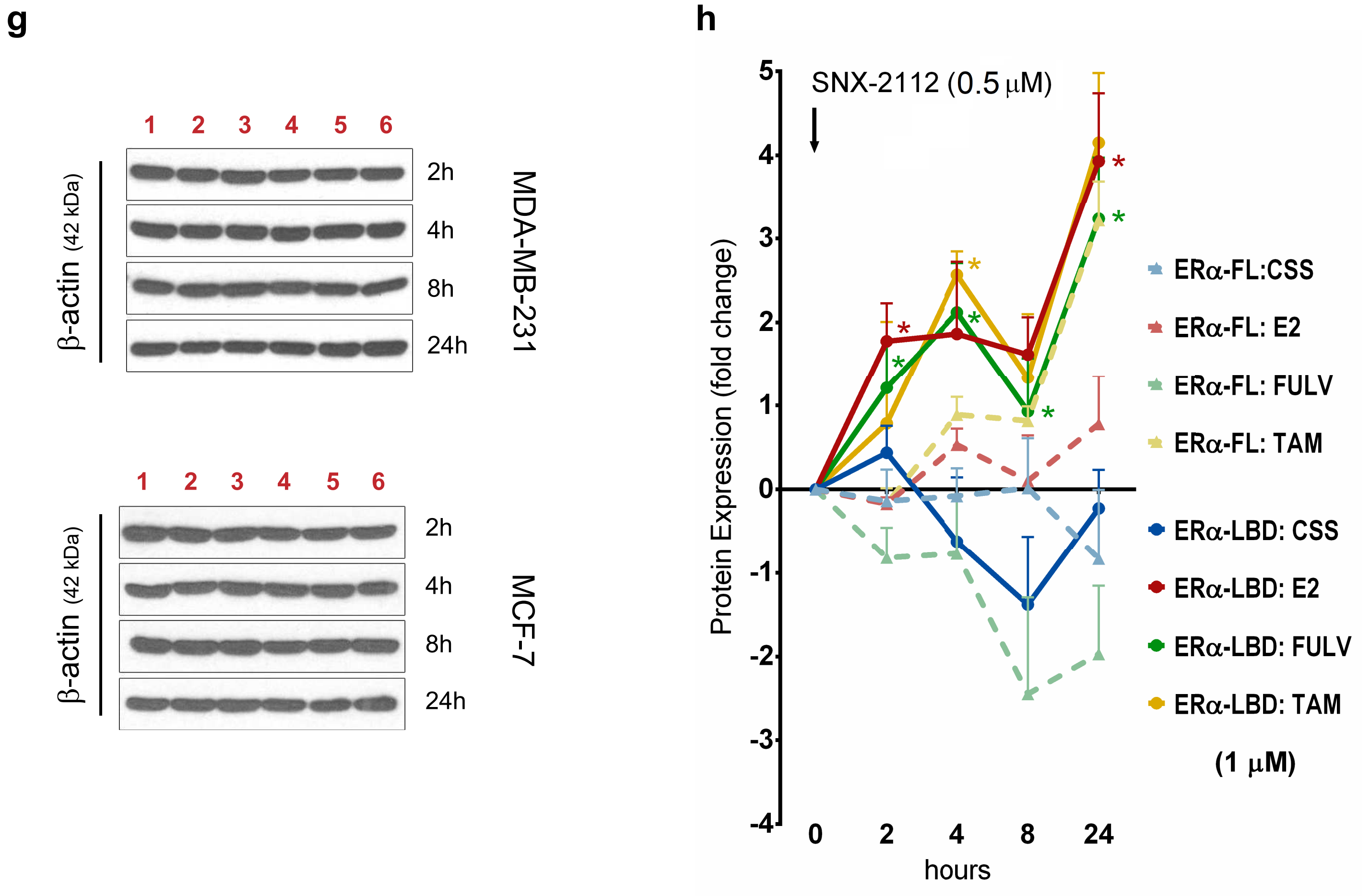
g
1 2 3 4 5 6
β-actin (42 kDa)
2h
4h
8h
24h
MDA-MB-231
1 2 3 4 5 6
β-actin (42 kDa)
2h
4h
8h
24h
MCF-7
h
SNX-2112 (0.5 μM)
Protein Expression (fold change)
hours
ERα-FL:CSS
ERα-FL: E2
ERα-FL: FULV
ERα-FL: TAM
ERα-LBD: CSS
ERα-LBD: E2
ERα-LBD: FULV
ERα-LBD: TAM
(1 μM)

Supplementary Figure 1. a Proliferation of different breast cancer (BC) cell lines, in the presence or absence of fulvestrant 1 µM treatment, assayed using resazurin reagent and expressed as fluorescence intensity (absorbance at 590 nm), taken as index of cell growth. Data are shown as mean ± s.e.m. (*n* = 3 independent experiments). *P* values are shown (two-way ANOVA, Sidak's test for multiple comparisons). FulvRes = fulvestrant resistant; vehicle = DMSO. **b** Western blot analysis of ERα protein expression in T-47D cells (Parental vs. FulvRes). Cells were cultured for 24 h in the presence of vehicle (DMSO) or fulvestrant 1 µM before lysis. ERα protein expression was normalized against β-actin. **c** Western blot analysis of ERα protein expression in breast BC cell lines. Cells were cultured for 24 h in the presence of vehicle (-) or fulvestrant 1 µM (+) before lysis. Vehicle = DMSO. ERα protein was detected by using an antibody raised against the C-terminal of ERα protein and expression was normalized against β-actin. Increasing levels of hormonal therapy resistance (HTR) are indicated. **d** Protein lysates from MCF-7 FulvRes and MDA-MB-231 (both treated with fulvestrant 1 µM for 24h) were immunoprecipitated using anti-ERα antibody. IP samples were run on two electrophoresis gels, one used for staining and band extraction, the other for WB check. Extracted bands were analyzed by capLC-MS/MS and results are shown. Sequenced/identified peptides from ERα protein are highlighted in dark grey and compared to the full-length ERα AA sequence. Different ERα domains with specific color code are also shown. AF1: transcription Activation Function-1 (cyan); DBD: DNA Binding Domain (red); Hinge (green); LBD/AF2: Ligand Binding Domain and transcription Activation Function-2 (yellow); amino acids involved in E2 binding (magenta); ERα-36 unique C-terminal (purple). **e** Analysis on ERα-LBD sequence. **f** As in (**c**). ERα protein was detected by using an antibody raised against the N-terminus of ERα protein. **g, h** ERα protein stability assay. ERα-FL and ERα-LBD protein levels were analyzed by western blot in MCF-7 and MDA-MB-231 cells, respectively. Protein samples were collected at different time points (2-24 h), after treatment. ERα protein levels were quantified and normalized against β-actin expression (**g**), and plotted as fold-change (log2), relative to SNX-2112 treatment alone (lane 2, medium containing physiological levels of E2 in pM range) (**h**). CSS = charcoal stripped serum. Data are presented as mean ± s.e.m. (*n* = 3 independent experiments); * *P* < 0.01, two-way ANOVA (Tukey's correction), ERα-LBD vs. ERα-FL.

Supplementary Figure 2

a

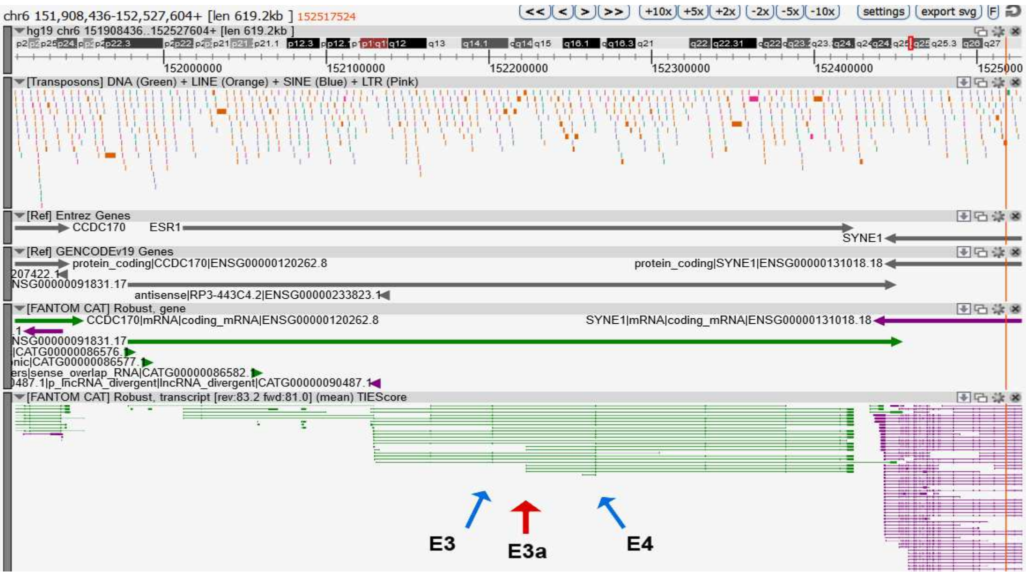

b

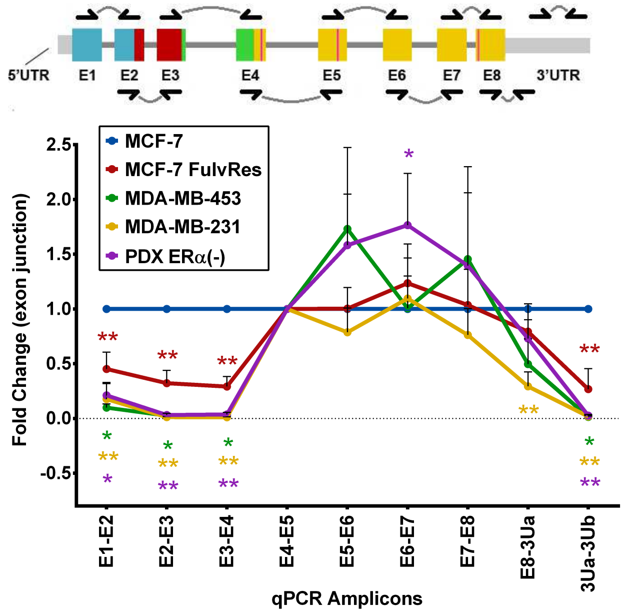

c

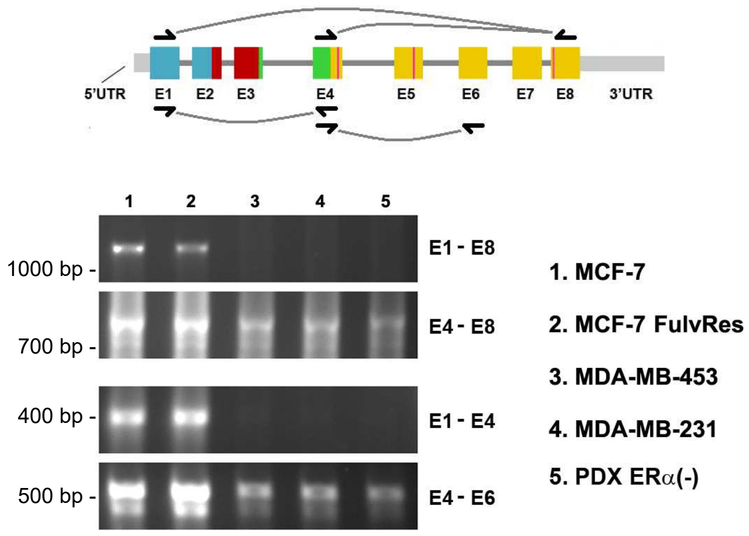

d

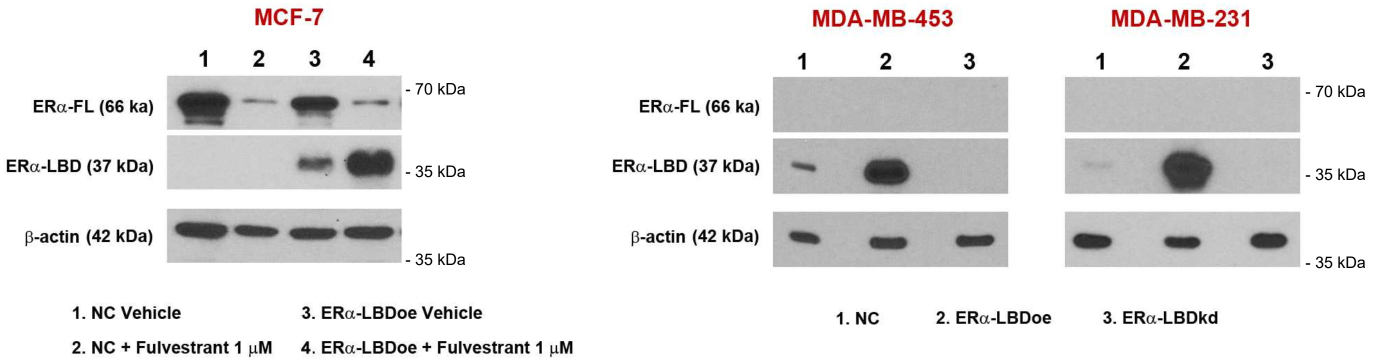

e

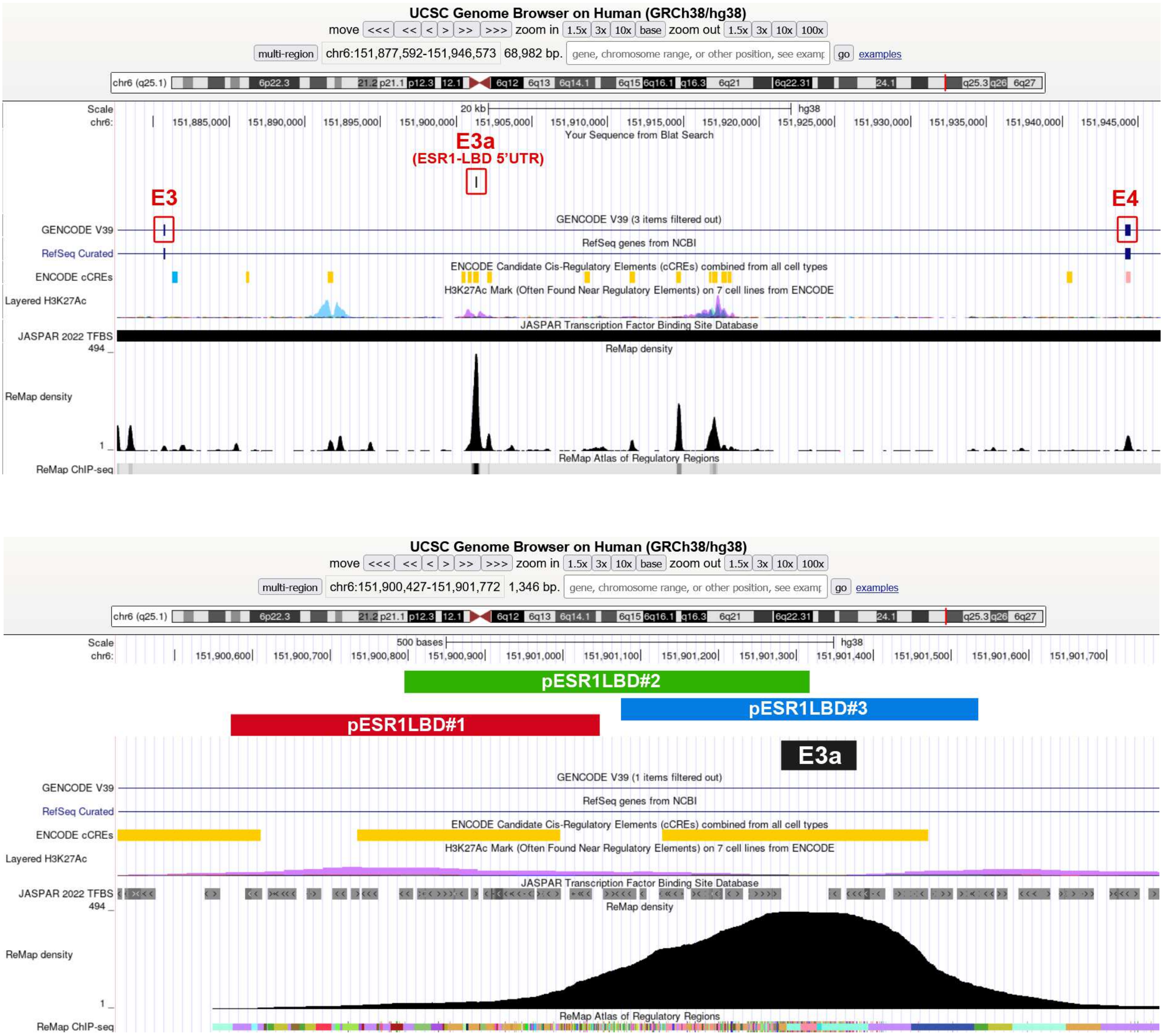
UCSC Genome Browser on Human (GRCh38/hg38)
chr6:151,877,592-151,946,573 68,982 bp.
E3a
(ESR1-LBD 5'UTR)
E3
E4
GENCODE V39
RefSeq Curated
ENCODE cCREs
Layered H3K27Ac
JASPAR 2022 TFBS
ReMap density
ReMap ChIP-seq
UCSC Genome Browser on Human (GRCh38/hg38)
chr6:151,900,427-151,901,772 1,346 bp.
pESR1LBD#2
pESR1LBD#3
pESR1LBD#1
E3a

f

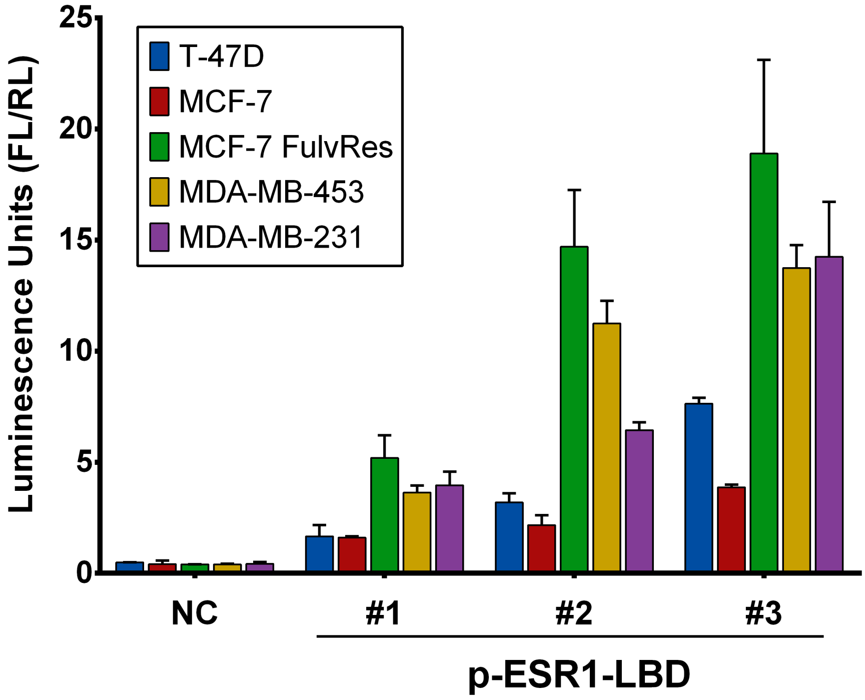
Luminescence Units (FL/RL)
25
20
15
10
5
0
T-47D
MCF-7
MCF-7 FulvRes
MDA-MB-453
MDA-MB-231
NC
#1
#2
#3
p-ESR1-LBD

g

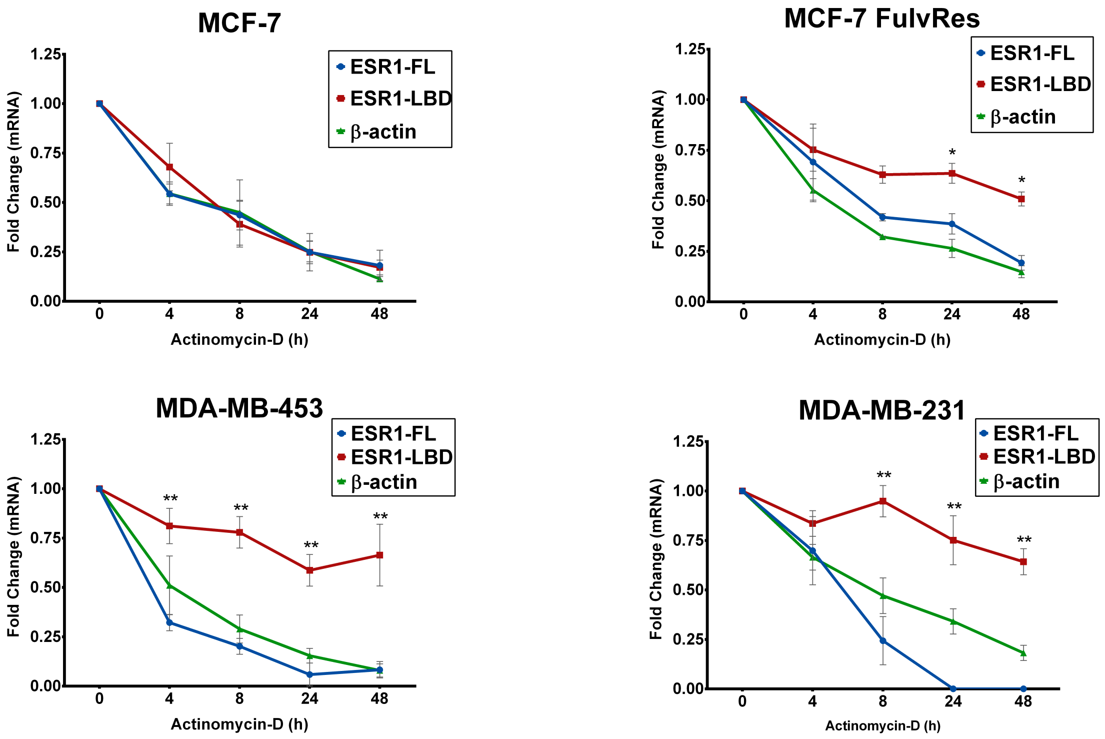
MCF-7
MCF-7 FulvRes
MDA-MB-453
MDA-MB-231
ESR1-FL
ESR1-LBD
β-actin
Fold Change (mRNA)
Actinomycin-D (h)
0.00
0.25
0.50
0.75
1.00
1.25
0
4
8
24
48
*
**

h

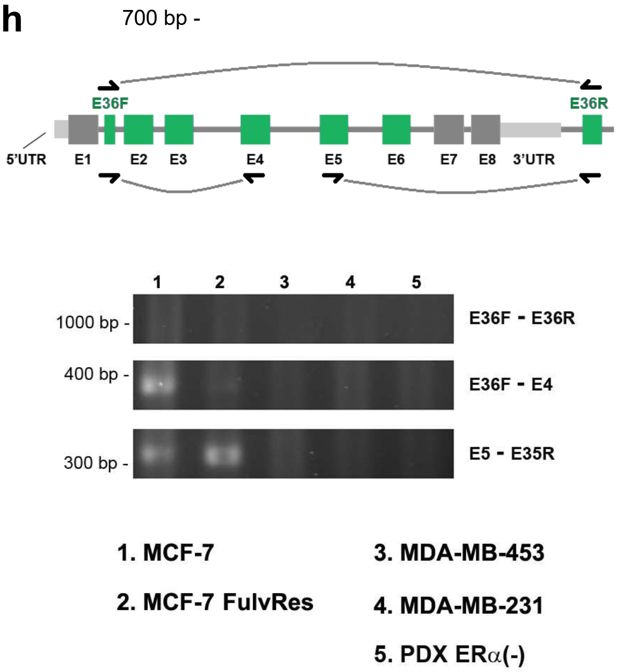
700 bp -
E36F
E36R
5'UTR
E1
E2
E3
E4
E5
E6
E7
E8
3'UTR
1
2
3
4
5
1000 bp -
400 bp -
300 bp -
E36F - E36R
E36F - E4
E5 - E35R
1. MCF-7
2. MCF-7 FulvRes
3. MDA-MB-453
4. MDA-MB-231
5. PDX ERα(-)

i

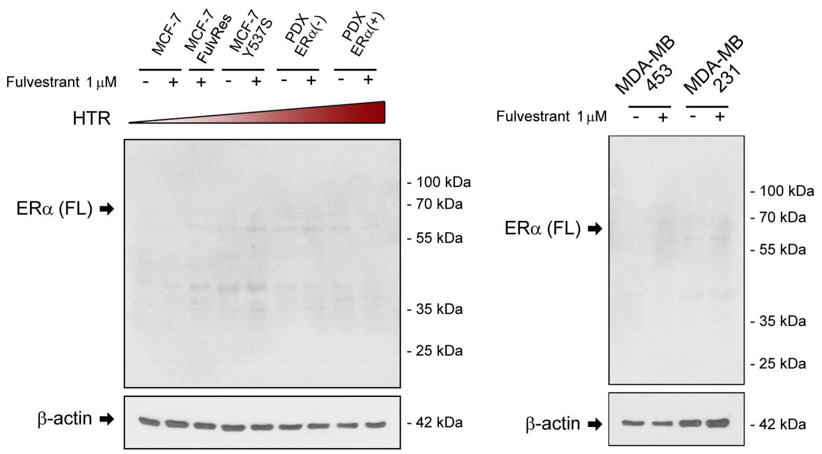
MCF-7
MCF-7 FulvRes
MCF-7 Y537S
PDX ERα(-)
PDX ERα(+)
Fulvestrant 1 μM
- + + - + - + - +
HTR
ERα (FL)
β-actin
- 100 kDa
- 70 kDa
- 55 kDa
- 35 kDa
- 25 kDa
- 42 kDa
MDA-MB 453
MDA-MB 231
Fulvestrant 1 μM
- + - +

Supplementary Figure 2. a Screenshot of ZEMBU Genome Browser showing data from FANTOM5/FANTOM CAT analyses collected on ESR1 gene. Exon 3 (E3) and exon 4 (E4) of ESR1 gene are highlighted by blue arrows, together with a new putative transcription starting site (TSS) mapped in the intronic region between the two exons (E3a, red arrow). **b** Expression of ESR1 mRNA exon junctions in BC cell lines, analyzed by qPCR. Plot shows expression fold change, normalized on RPLP0 and relative to MCF-7 sample. Color-code of exons is based on different ERα protein domain. Data are presented as mean ± s.e.m. (n = 3 independent experiments). * $P < 0.05$, ** $P < 0.01$, two-way ANOVA (Fisher's LSD test). **c** Expression of different ESR1 mRNA regions in BC cell lines, analyzed by RT-PCR. The scheme above depicts PCR amplicons and their position on ESR1 transcript. Color-code of exon as in (**b**). **d** Western blot analysis of ERα protein variants expression (ERα-FL vs. ERα-LBD) in different BC cell lines (stable clones). Vehicle (DMSO 0.01%) or fulvestrant treatment (1 µM, 24 h) was added to MCF-7 cells (on the left). NC: cells transduced with control vector. ERα-LBDoe: cells transduced with ESR1-LBD CDS sequence (exon 4 to exon 8), inducing ERα-LBD protein overexpression. ERα-LBDkd: cells transduced with CRISPR/CAS9 vector targeting ESR1 exon 4 and promoting ERα-LBD protein knockdown. **e** Screenshots of UCSC Genome Browser showing regulatory elements and transcription binding site mapping on ESR1 exon E3a genomic region (putative ESR1-LBD 5'UTR). The zoomed area below shows three different genomic DNA regions representing putative ESR1-LBD promoter (pESR1-LBD) and screened for transcriptional activity. **f** pESR1-LBD promoter-driven luciferase reporter assay in transfected T-47D, MCF-7, MCF-7 FulvRes, MDA-MB-453 and MDA-MB-231 cells, respectively. Firefly luciferase (FL) levels were normalized to Renilla luciferase (RL). Controls (NC) = cells transfected with empty pGL3.basic plasmid. Data are presented as mean ± s.e.m. (n = 2 independent experiments). **g** BC cells lines were treated with actinomycin-D and tested by qPCR at different time points for ESR1 and β-actin mRNA expression. ESR1-FL: PCR amplicon ranging from ESR1 exon E1 to E3. ESR1-LBD: PCR amplicon ranging from ESR1 exon E4 to E5. β-actin transcript was taken as positive control for mRNA decay. Plots shows mRNA fold change, normalized on RPLP0 and relative to time 0. Data are presented as mean ± s.e.m. (n = 3 independent experiments). * $P < 0.05$, ** $P < 0.01$, two-way ANOVA (Fisher's LSD test). **h** Expression of different ESR1 mRNA regions in BC cell lines, analyzed by RT-PCR. The scheme above depicts PCR amplicons and their position on ESR1 transcript. Exons of ERα-36 transcript are in green. **i** Western blot analysis of ERα-36 protein expression in BC cell lines. Cells were cultured for 24 h in the presence of vehicle (-) or fulvestrant 1 µM (+) before lysis.

Vehicle = DMSO. ERα protein expression was normalized against β-actin. Increasing levels of hormonal therapy resistance (HTR) are indicated.

Supplementary Figure 3

a

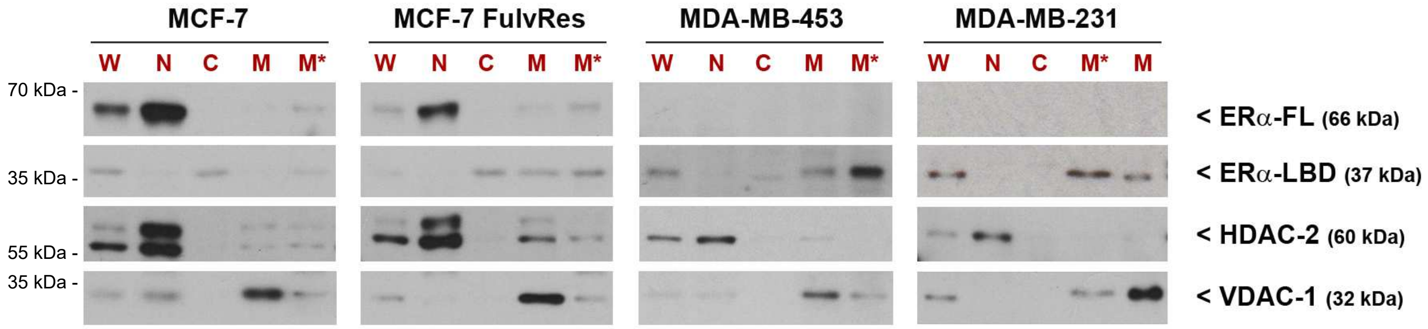

W = whole lysate
N = Nuclear fraction
C = Cytosolic fraction
M = Mitochondrial fraction
M* = Mitochondrial fraction + proteinase K

b

Anti-ERa (Cell Signaling)	MCF-7		MCF-7 +Fulv		MCF-7 FulvRes +Fulv	
	Nucl	Mito	Nucl	Mito	Nucl	Mito
threshold A	50	50	50	50	50	50
threshold B	50	50	50	50	50	50
number of colocalized voxels	535701	28128	75341	39987	49694	96212
% of dataset colocalized	1.7	0.09	0.27	0.14	0.23	0.44
% of ROI colocalized	1.7	0.09	0.27	0.14	0.23	0.44
% of volume A above threshold colocalized (DAPI / OXPHOS)	34.71	4.4	29.1	10.63	30.14	46.75
**% of volume B above threshold colocalized (ERa)**	53.89	2.83	31.28	16.6	13.55	26.24
% of material A above threshold colocalized (DAPI / OXPHOS)	35.48	4.91	29.52	11.89	29.24	50.07
**% of material B above threshold colocalized (ERa)**	56.09	2.06	34.49	14.63	14.94	25.42
% of ROI material A colocalized (DAPI / OXPHOS)	10.77	2.85	7.46	8.42	7.66	37.34
**% of ROI material B colocalized (ERa)**	31.29	1.15	15.58	6.61	9.89	16.82
Pearson's coefficient in dataset volume	0.5687	0.0975	0.5852	0.3261	0.452	0.4067
Pearson's coefficient in ROI volume	0.5687	0.0975	0.5852	0.3261	0.452	0.4067
Pearson's coefficient in colocalized volume	-0.0016	-0.1769	0.0433	-0.0152	-0.0603	0.0343
original Mander's coefficient A	0.7252	0.8992	0.7513	0.9647	0.6997	0.9805
original Mander's coefficient B	0.996	0.4609	0.9714	0.6228	0.9063	0.5377
thresholded Mander's coefficient A	0.1586	0.0551	0.145	0.1061	0.2041	0.4425
thresholded Mander's coefficient B	0.4157	0.0532	0.2268	0.1976	0.132	0.2136

Anti-ERa (Cell Signaling)	MDA-MB-453		MDA-MB-231		PDX ERα(-)	
	Nucl	Mito	Nucl	Mito	Nucl	Mito
threshold A	50	50	50	50	50	50
threshold B	50	50	50	50	50	50
number of colocalized voxels	2634	90200	23806	81216	4412	78345
% of dataset colocalized	0.01	0.3	0.11	0.37	0.02	0.31
% of ROI colocalized	0.01	0.3	0.11	0.37	0.02	0.31
% of volume A above threshold colocalized (DAPI / OXPHOS)	3.77	33.87	6.65	45.95	2.79	31.79
**% of volume B above threshold colocalized (ERa)**	0.67	22.8	10.9	37.19	2.16	38.28
% of material A above threshold colocalized (DAPI / OXPHOS)	3.77	39.11	6.43	53.84	2.73	35.49
**% of material B above threshold colocalized (ERa)**	0.53	22.47	9.27	41.03	1.8	38.27
% of ROI material A colocalized (DAPI / OXPHOS)	0.31	23.7	4.55	40.54	0.61	23.12
**% of ROI material B colocalized (ERa)**	0.26	11.13	3.66	16.19	0.79	16.89
Pearson's coefficient in dataset volume	0.2851	0.4097	0.3069	0.5341	0.3525	0.5341
Pearson's coefficient in ROI volume	0.2851	0.4097	0.3069	0.5341	0.3525	0.5341
Pearson's coefficient in colocalized volume	-0.0453	0.0104	-0.0515	0.1891	-0.0215	-0.0183
original Mander's coefficient A	0.7753	0.9737	0.9518	0.9927	0.7565	0.9925
original Mander's coefficient B	0.9236	0.6657	0.7661	0.5258	0.9522	0.8155
thresholded Mander's coefficient A	0.0735	0.3069	0.0774	0.4472	0.0621	0.2852
thresholded Mander's coefficient B	0.0127	0.1644	0.1685	0.2169	0.0576	0.2711

c

ERα staining with C-terminal antibody (D8H8)

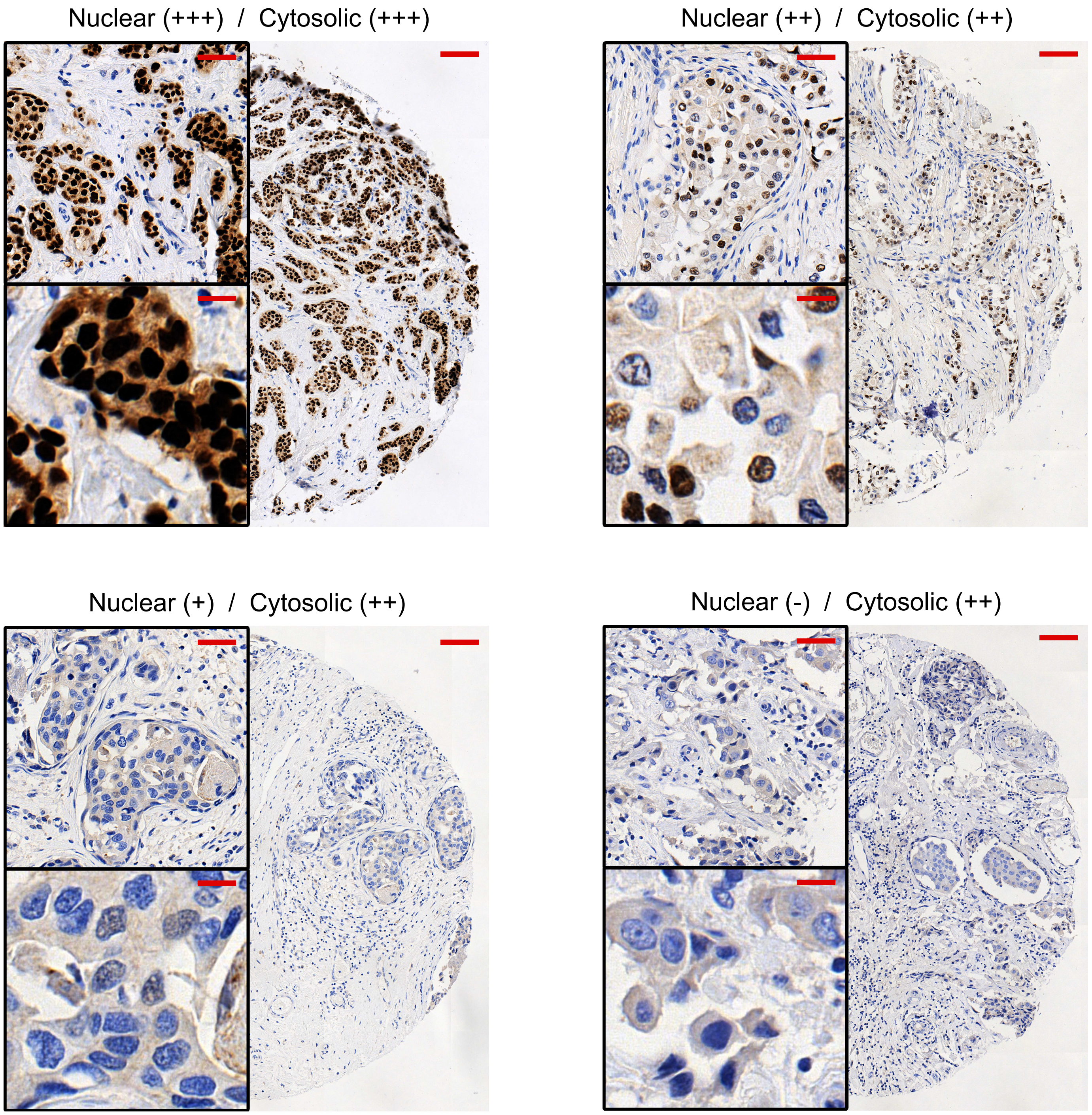

d

ERα staining with N-terminal antibody (6F11)

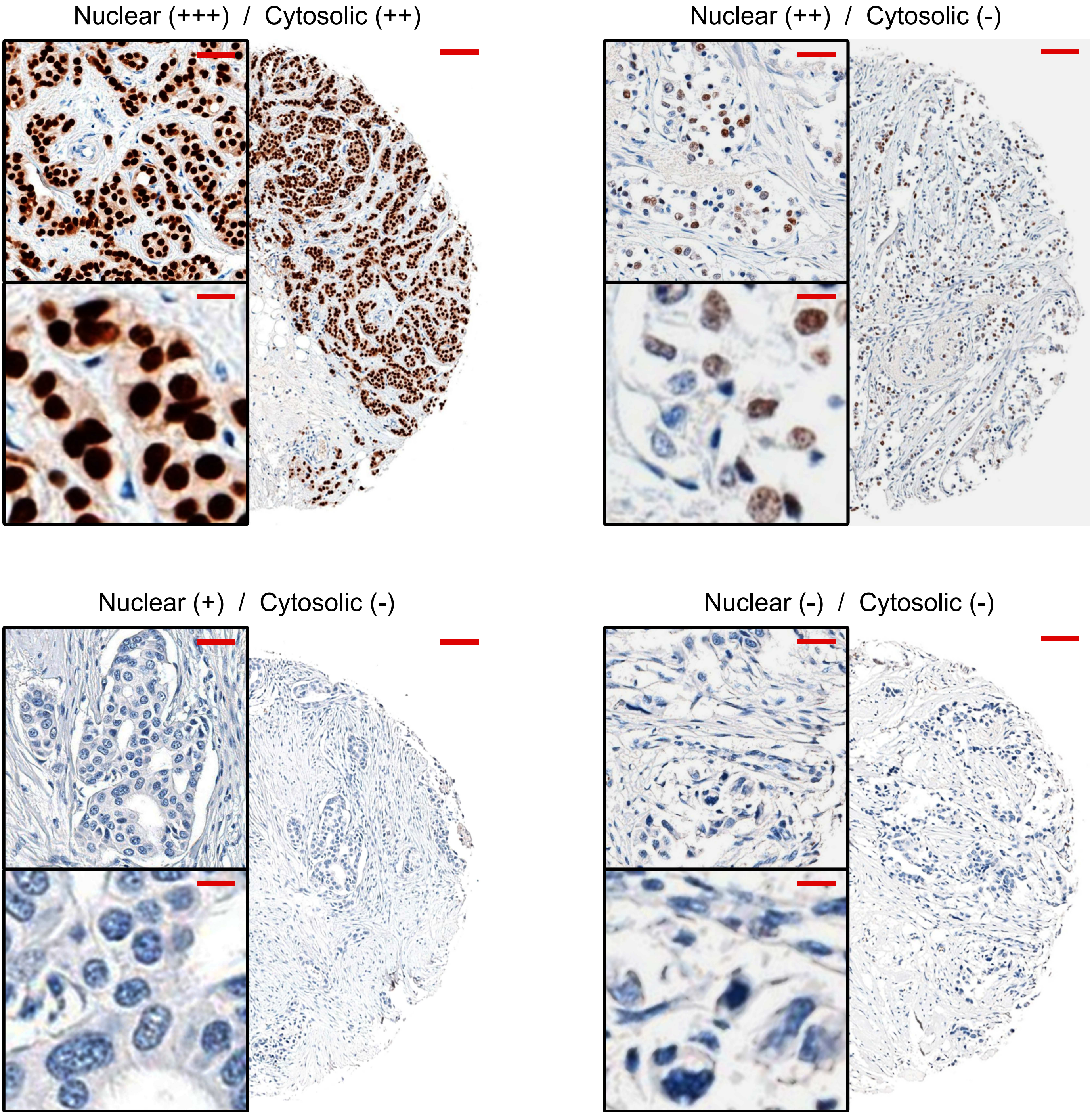

e **ERα staining with C-terminal antibody (D8H8) - Cytosolyc Grading**

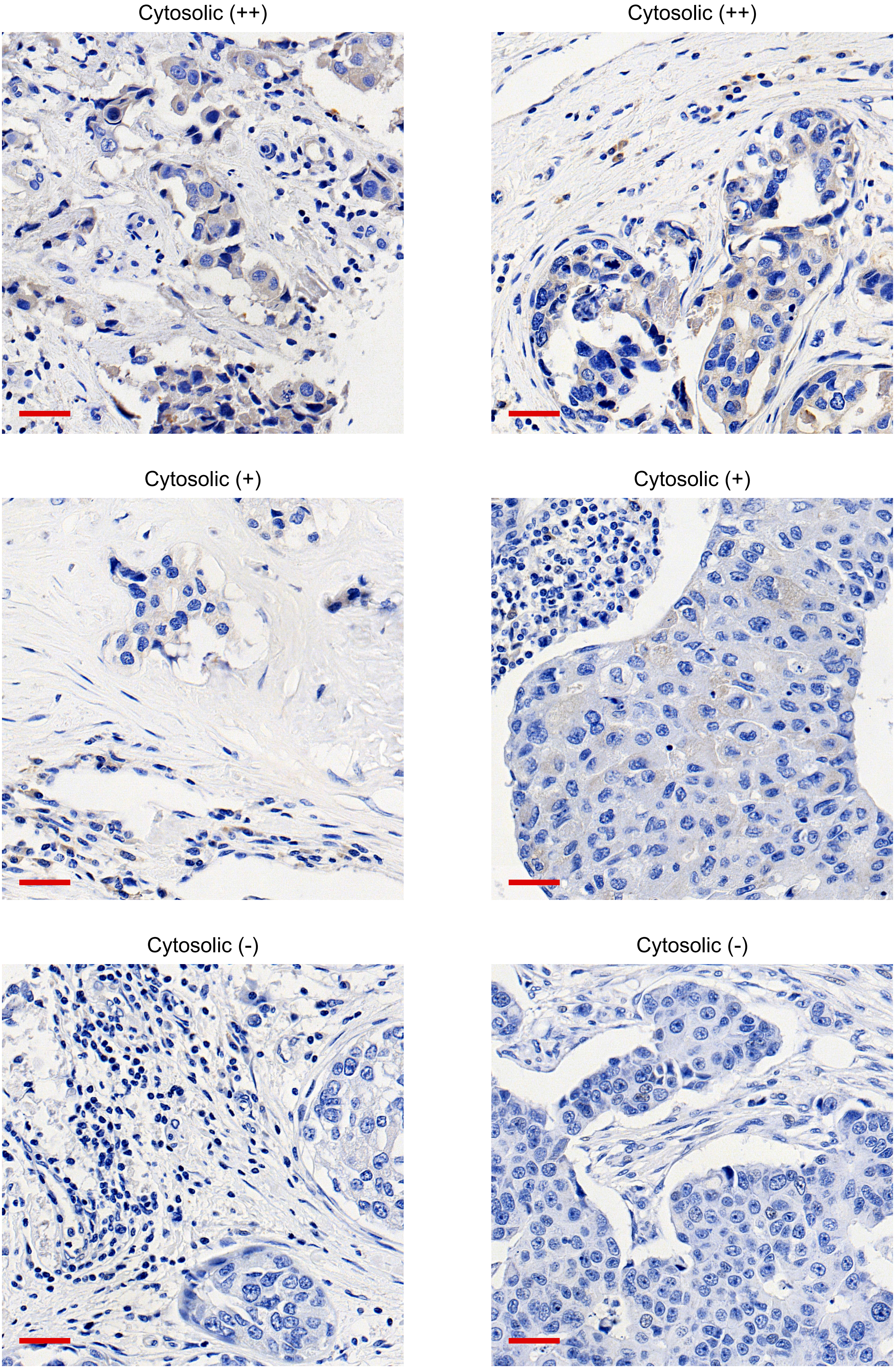

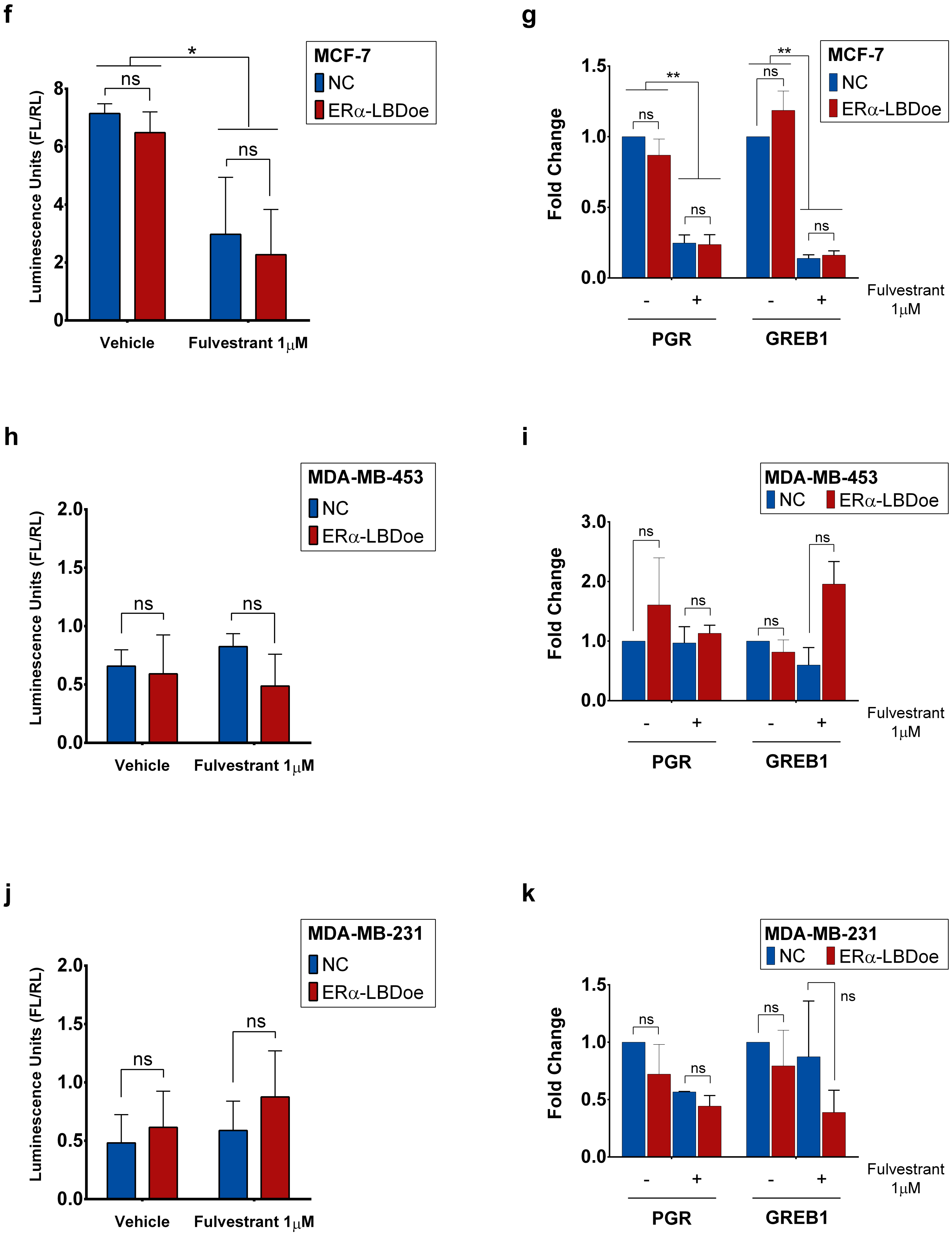
f
MCF-7
NC
ERα-LBDoe
Luminescence Units (FL/RL)
8
6
4
2
0
*
ns
ns
Vehicle
Fulvestrant 1μM
g
MCF-7
NC
ERα-LBDoe
Fold Change
1.5
1.0
0.5
0.0
**
**
ns
ns
ns
ns
-
+
-
+
Fulvestrant 1μM
PGR
GREB1
h
MDA-MB-453
NC
ERα-LBDoe
Luminescence Units (FL/RL)
2.0
1.5
1.0
0.5
0.0
ns
ns
Vehicle
Fulvestrant 1μM
i
MDA-MB-453
NC
ERα-LBDoe
Fold Change
3.0
2.0
1.0
0.0
ns
ns
ns
ns
-
+
-
+
Fulvestrant 1μM
PGR
GREB1
j
MDA-MB-231
NC
ERα-LBDoe
Luminescence Units (FL/RL)
2.0
1.5
1.0
0.5
0.0
ns
ns
Vehicle
Fulvestrant 1μM
k
MDA-MB-231
NC
ERα-LBDoe
Fold Change
1.5
1.0
0.5
0.0
ns
ns
ns
ns
-
+
-
+
Fulvestrant 1μM
PGR
GREB1

Supplementary Figure 3. a Western Blot analysis of ERα protein variants and their expression in BC cell fractions. HDAC2 was taken as nuclear protein marker whereas VDAC1 was taken as mitochondrial protein marker. Samples from mitochondrial fractions were also treated with proteinase K (50 µg/ml). **b** Table summarizing statistics from confocal analysis on different BC cell lines. In particular, colocalization data are shown. Blue and red numbers indicate colocalization level of ERα protein into the nucleus or mitochondria of cells, respectively. **c, d** Representative images of ERα staining by immunohistochemistry (IHC) in breast cancer tissue microarray (TMA). Two different ERα antibodies were used: D8H8 (against the C-terminal of the protein) (**c**) and 6F11 (against the N-terminal of the protein) (**d**). All histological sections were counterstained with hematoxylin. Scale bars and magnification: 160 µm (15X); 40 µm (63X); 10 µm (250X). **e** Representative images of ERα cytosolic staining by IHC in BC-TMA by using ERα D8H8 antibody. Different grading is described (++ vs. + vs. -). All histological sections were counterstained with hematoxylin. Scale bar and magnification: 40 µm (63X). **f, h, j** ERE (Estrogen Responsive Element) promoter-driven luciferase reporter assay in MCF-7, MDA-MB-453 and MDA-MB-231 cells, respectively. Firefly luciferase levels were normalized to Renilla luciferase. Controls (NC) were compared to cells overexpressing ERα-LBD (oe), either in the presence or absence of fulvestrant 1 µM treatment (24 h). Vehicle = DMSO 0.01%. Data are presented as mean ± s.e.m. (n = 3 independent experiments). * $P < 0.05$, ns = not significant; two-way ANOVA (Sidak's correction). **g, i, k** Expression of PGR and GREB1 mRNA in MCF-7, MDA-MB-453 and MDA-MB-231 cells, respectively. Analysis was carried out by qPCR. Samples and treatments: same as above. Plot shows fold change expression, relative to NC without treatment. In all panels, blue indicates NC samples and red indicates ERα-LBDoe/kd samples. Data are presented as mean ± s.e.m. (n = 6 or n = 4 independent experiments). ** $P < 0.01$, ns = not significant; two-way ANOVA (Sidak's correction).

Supplementary Figure 4

a

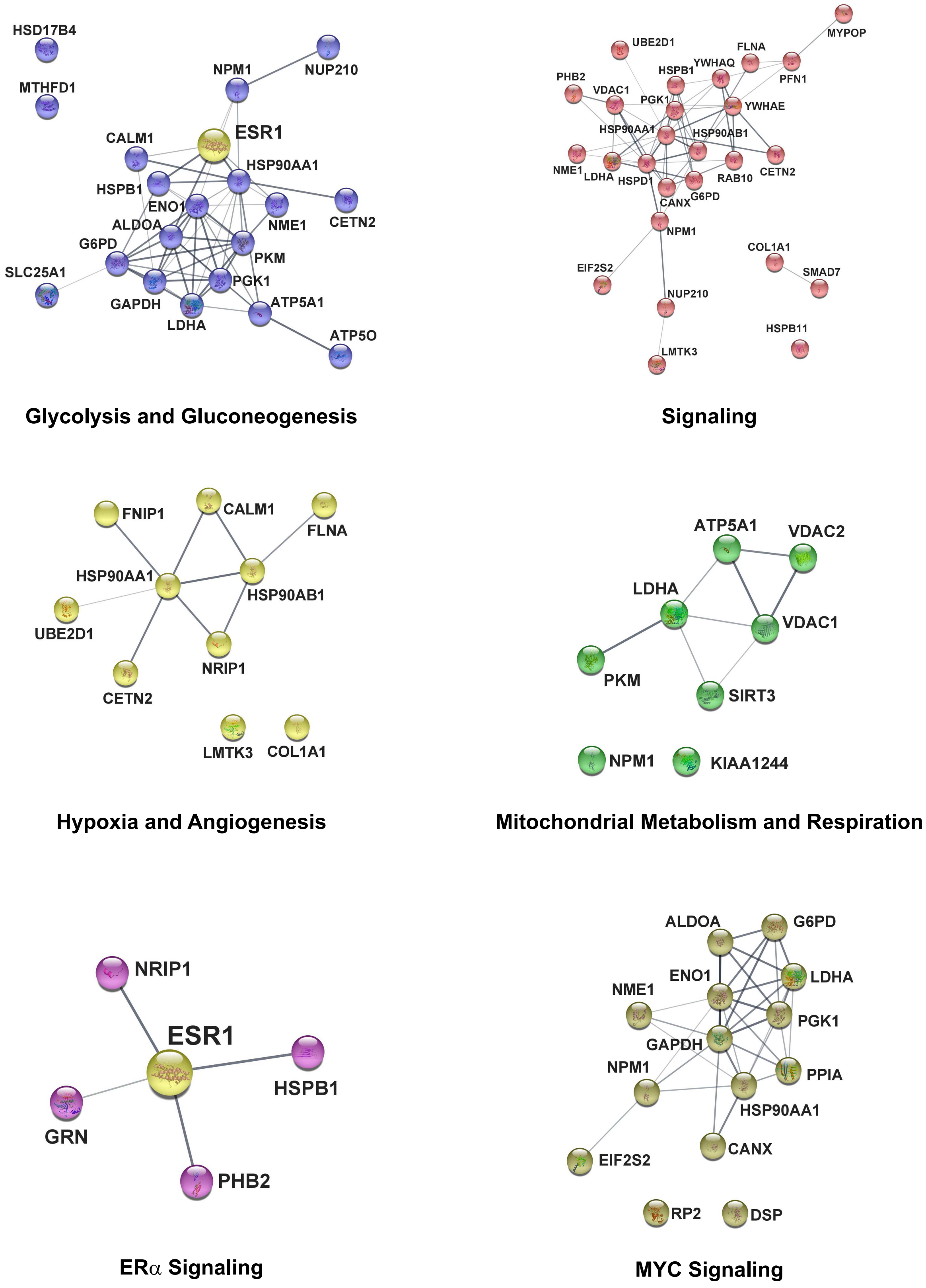

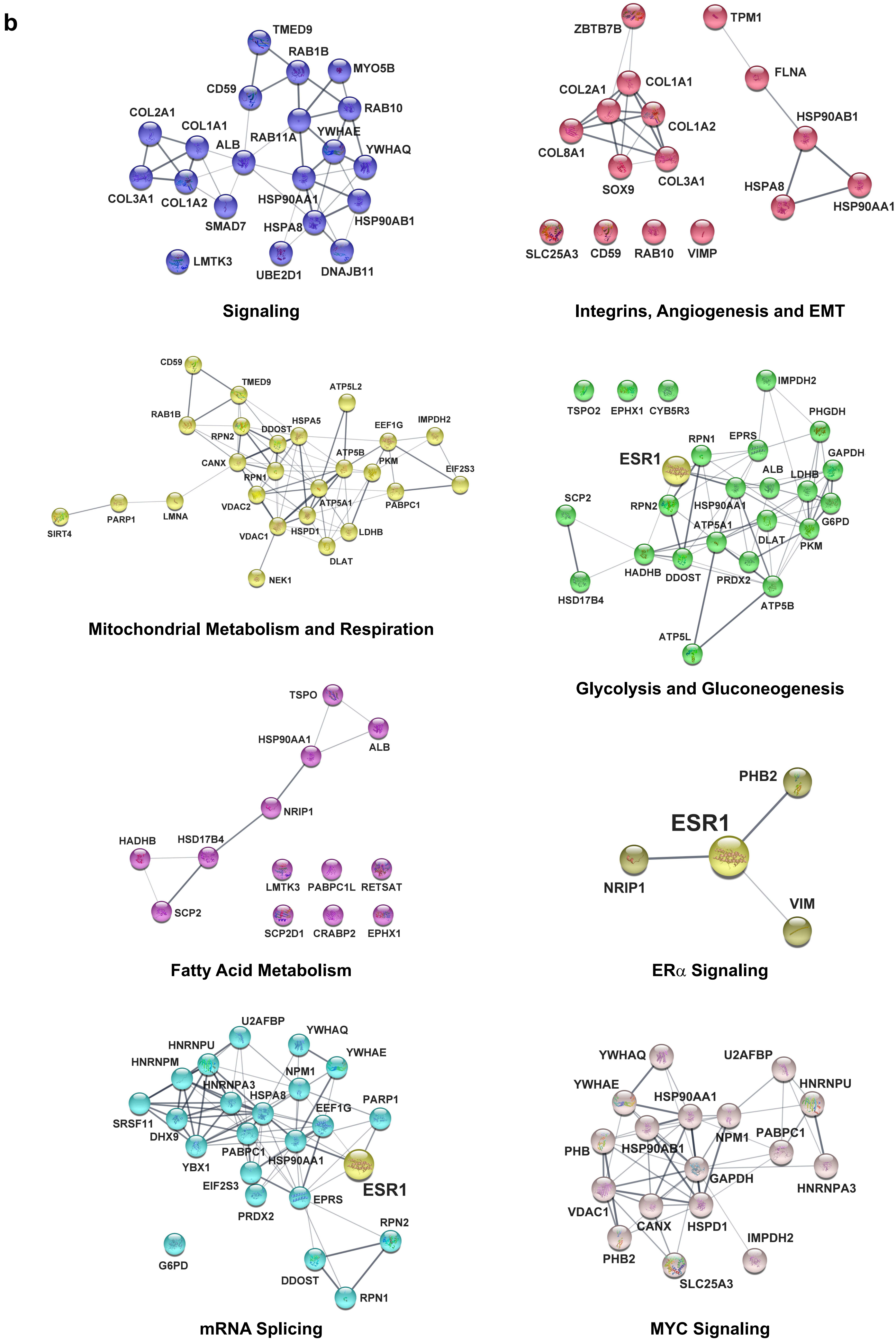
b
TMED9
RAB1B
MYO5B
CD59
RAB10
COL2A1
COL1A1
ALB
RAB11A
YWHAE
YWHAQ
COL3A1
COL1A2
SMAD7
HSP90AA1
HSPA8
HSP90AB1
LMTK3
UBE2D1
DNAJB11
Signaling
ZBTB7B
TPM1
FLNA
COL2A1
COL1A1
COL1A2
COL8A1
SOX9
COL3A1
HSP90AB1
HSPA8
HSP90AA1
SLC25A3
CD59
RAB10
VIMP
Integrins, Angiogenesis and EMT
CD59
TMED9
ATP5L2
RAB1B
HSPA5
EEF1G
IMPDH2
RPN2
DDOST
ATP5B
CANX
PKM
EIF2S3
RPN1
VDAC2
ATP5A1
PABPC1
SIRT4
PARP1
LMNA
VDAC1
HSPD1
LDHB
DLAT
NEK1
Mitochondrial Metabolism and Respiration
TSPO2
EPHX1
CYB5R3
IMPDH2
PHGDH
RPN1
EPRS
ESR1
GAPDH
ALB
LDHB
SCP2
RPN2
HSP90AA1
ATP5A1
G6PD
DLAT
PKM
HADHB
DDOST
PRDX2
HSD17B4
ATP5B
ATP5L
Glycolysis and Gluconeogenesis
TSPO
HSP90AA1
ALB
NRIP1
HADHB
HSD17B4
SCP2
LMTK3
PABPC1L
RETSAT
SCP2D1
CRABP2
EPHX1
Fatty Acid Metabolism
PHB2
ESR1
NRIP1
VIM
ERα Signaling
U2AFBP
YWHAQ
HNRNPU
YWHAE
HNRNPM
NPM1
HNRNPA3
HSPA8
EEF1G
PARP1
SRSF11
DHX9
PABPC1
YBX1
HSP90AA1
EIF2S3
ESR1
EPRS
PRDX2
RPN2
G6PD
DDOST
RPN1
mRNA Splicing
YWHAQ
U2AFBP
YWHAE
HNRNPU
HSP90AA1
NPM1
PABPC1
PHB
HSP90AB1
GAPDH
HNRNPA3
VDAC1
CANX
HSPD1
IMPDH2
PHB2
SLC25A3
MYC Signaling

Supplementary Figure 4. a, b Analysis of ERα-LBD protein-protein interaction (PPI) network in MCF-7 and TNBC models respectively, using Cytoscape and STRING software. Proteins are presented as nodes. PPIs networks associated to specific biological functions are presented by using different colors.

Supplementary Figure 5

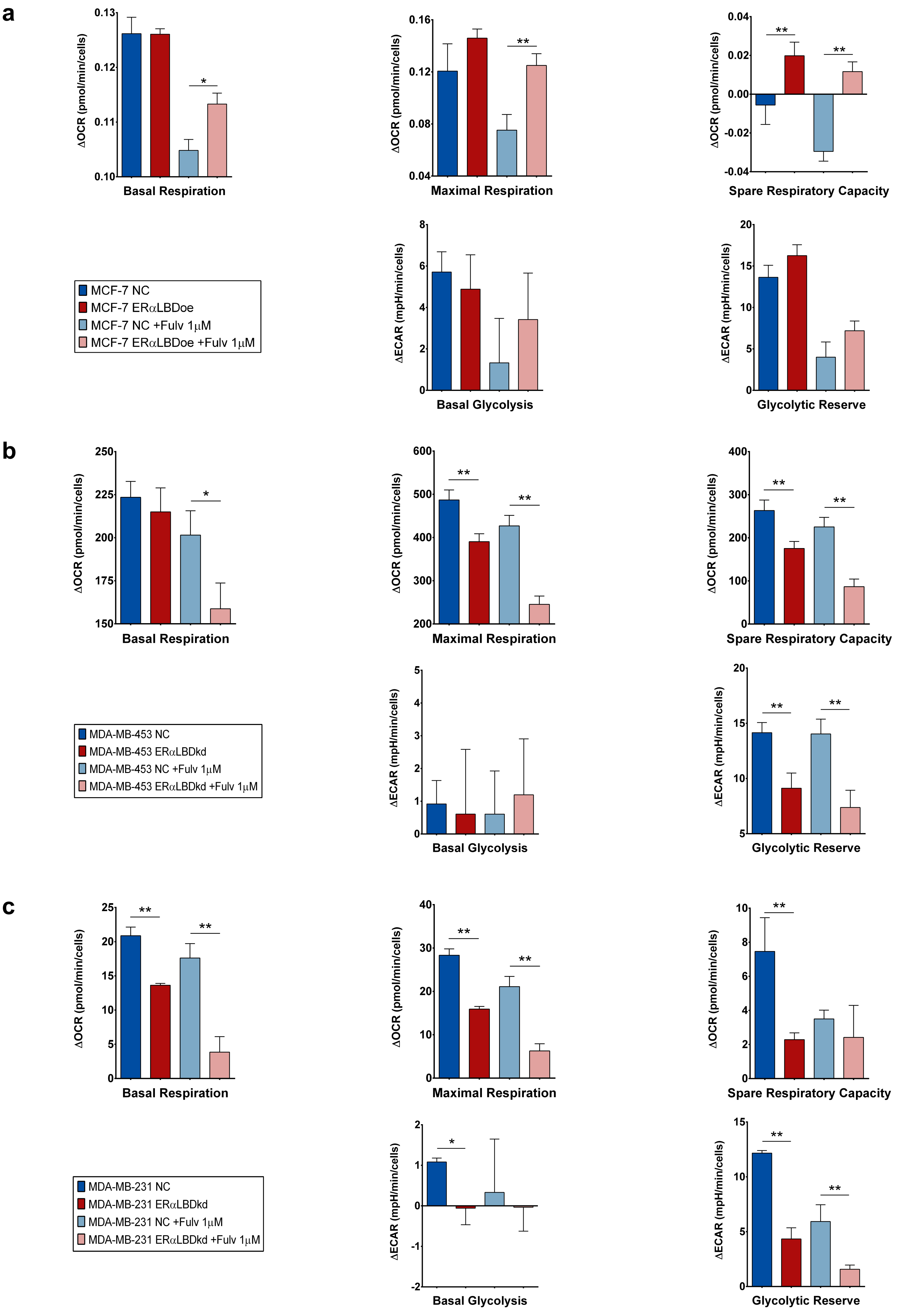

Supplementary Figure 5. a Evaluation of respiratory and glycolytic parameters in MCF-7 cell clones. Fulvestrant 1 µM (24 h pre-treatment) or vehicle (DMSO) was added to cells. ERα-LBD overexpressing (oe) cells were compared to controls (NC). Analyses were carried out by using XF Cell Mito Stress Kit (Agilent). Following manufacturer's guidelines, the calculation of all parameters was based on ΔOCR and ΔECAR values collected during the assay. **b, c** Evaluation of respiratory and glycolytic parameters in TNBC cells (MDA-MB-453/-231). Fulvestrant 1 µM (24 h pre-treatment) or vehicle (DMSO) was added to cells. Cells with ERα-LBD knockdown (kd) were compared to controls (NC). Analysis was carried out as described above. Data in the figures are presented as mean ± s.e.m. (n = 2 independent experiments). * $P < 0.05$, ** $P < 0.01$; unpaired t test, one-sided.

Supplementary Figure 6

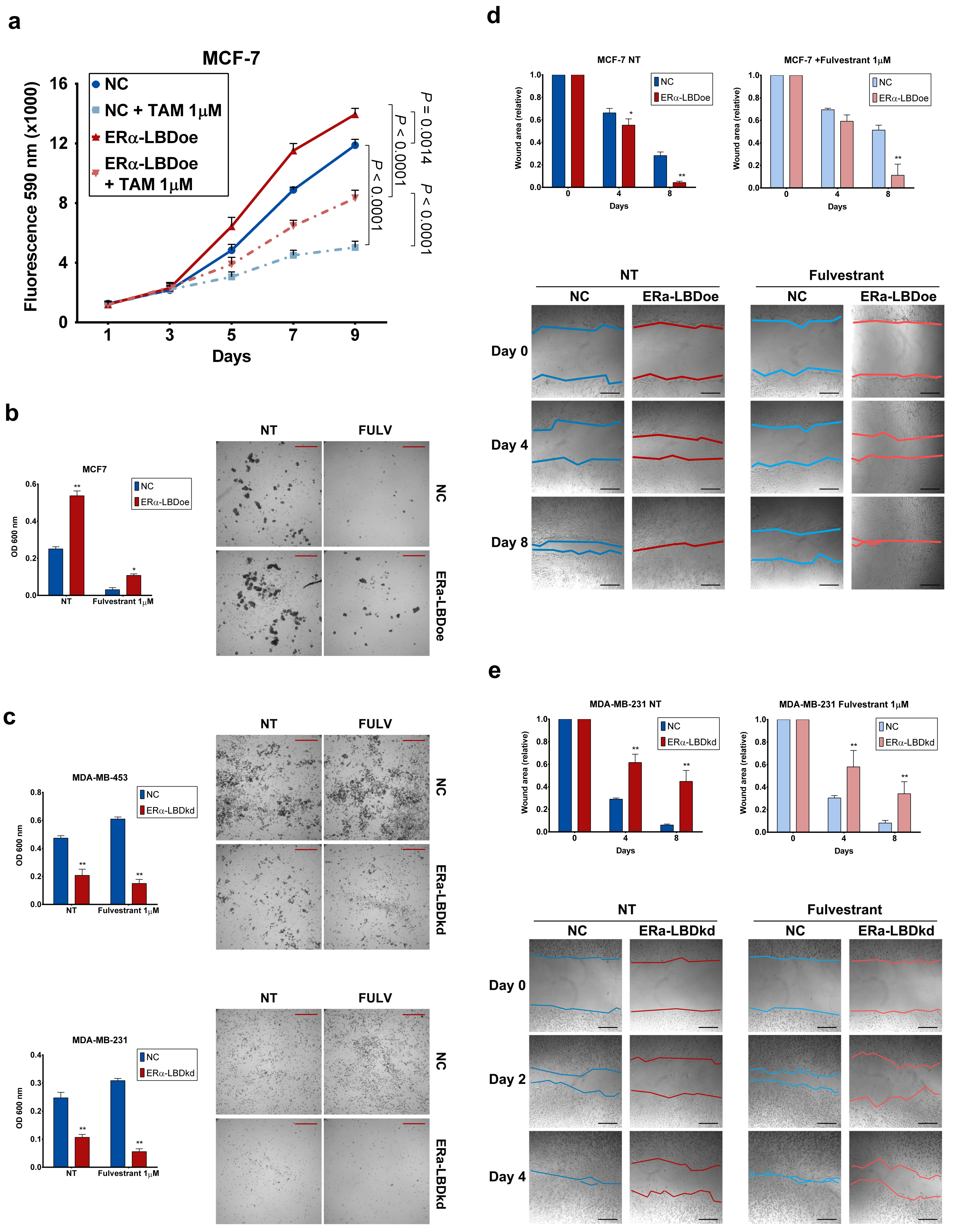

Supplementary Figure 6. a *In vitro* proliferation of MCF-7 cell clones, using resazurin reagent and expressed as fluorescence intensity (absorbance at 590 nm). ERα-LBD overexpression was compared to controls (NC) and cell proliferation was tested in the presence or absence of tamoxifen 1 µM treatment (n = 3 independent experiments). All P values describing statistical differences between samples are shown. **b, c** 3-D growth of BC stable clones, measured as optical density (OD) at 600 nm of cell suspensions. ERα-LBD overexpression (MCF-) or knockdown (MDA-MB-453 & 231) was compared to controls (NC), either in the absence (NT) or presence of fulvestrant 1 µM treatment. Representative images of cell 3-D growth are also shown. Fulv = fulvestrant 1 µM. Scale bar: 200 µm. Magnification: 10X. **d, e** Cell migration of BC stable clones was tested by wound healing assay, at different time points (days). Samples and treatments: same as in (**b**). In all panels, blue indicates NC samples and red indicates ERαLBDoe/kd samples. Representative images of scratches are also shown, with wound edges highlighted by different colored lines. Fulv = fulvestrant 1 µM. Scale bar: 200 µm. Magnification: 10X. All data in the figure are presented as mean ± s.e.m. (n = 3 independent experiments), * $P < 0.05$, ** $P < 0.01$, two-way ANOVA (Sidak's correction).

Supplementary Figure 7

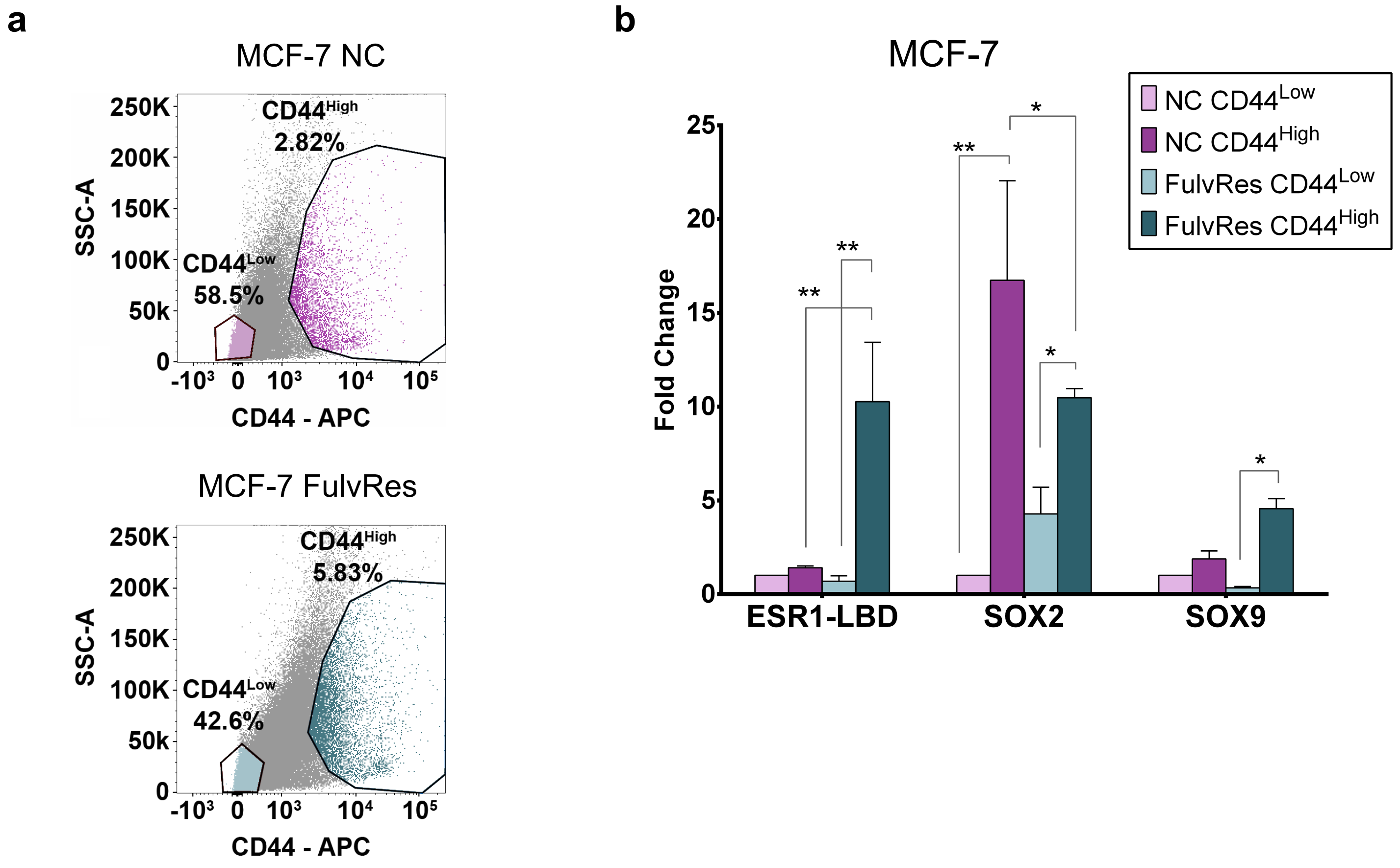

Supplementary Figure 7. a Representative images of flow cytometry analysis of CD44^{Low} and CD44^{High} cells, isolated from BC murine xenografts models (MCF-7 and MCF-7 FulvRes). Color code: purple, MCF-7 NC; teal, MCF-7 FulvRes; light, CD44^{Low}; dark, CD44^{High}. **b** Total RNA was extracted from CD44$^{Low/High}$ sorted BC cells and analyzed by qPCR. Plot shows levels of ESR1-LBD (normalized first on RPLP0, then on ESR1-FL) and stem-cell markers Sox2 and Sox9 (normalized on RPLP0), expressed as fold change (CD44^{High} vs. CD44^{Low}). Color code as in (**a**). Data are presented as mean ± s.e.m. (*n* = 2 independent experiments; *n* = 3 replicates, each experiment). * $P < 0.05$, ** $P < 0.01$, two-way ANOVA (Fisher's LSD test) and unpaired t test, two-tailed.

Supplementary Figure 8

a

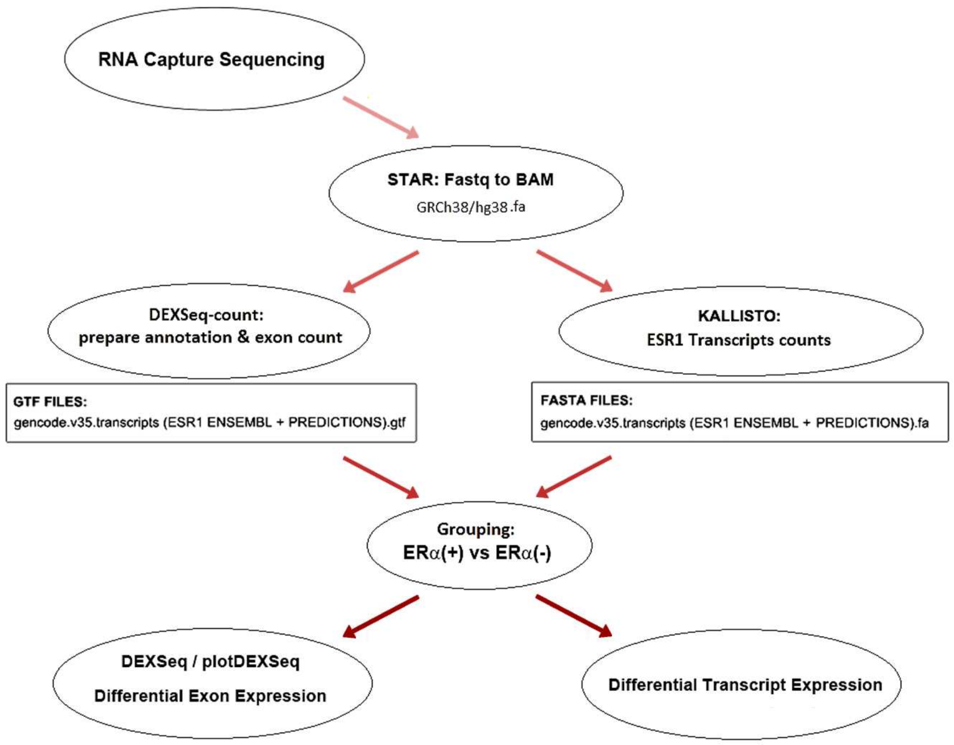

b

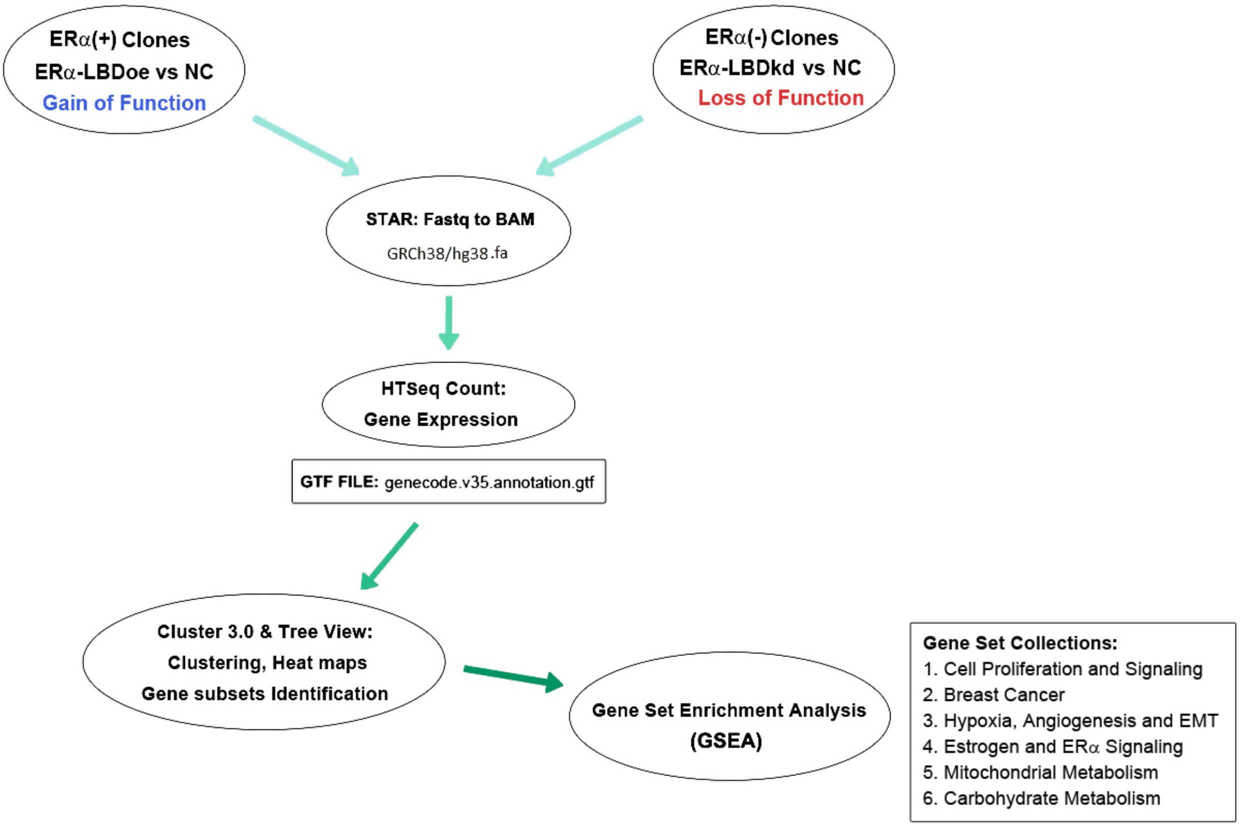

Supplementary Figure 8. a Schematic summary of the pipeline used to analyze raw data obtained from RNA capture-sequencing experiment. **b** Schematic summary of the pipeline used to analyze raw data from RNA sequencing experiment on BC cell clones.
